# E2F1-Mediated Induction of NFYB Attenuates Apoptosis via Joint Regulation of a Pro-Survival Transcriptional Program

**DOI:** 10.1371/journal.pone.0127951

**Published:** 2015-06-03

**Authors:** Xiaolei Jiang, Joseph Roy Nevins, Igor Shats, Jen-Tsan Chi

**Affiliations:** 1 Duke Center for Genomic and Computational Biology, Duke University, Durham, North Carolina, United States of America; 2 Department of Molecular Genetics and Microbiology, Duke University, Durham, North Carolina, United States of America; 3 Department of Biomedical Engineering, Duke University Medical Center, Durham, North Carolina, United States of America; University Hospital of Navarra, SPAIN

## Abstract

The E2F1 transcription factor regulates cell proliferation and apoptosis through the control of a considerable variety of target genes. Previous work has detailed the role of other transcription factors in mediating the specificity of E2F function. Here we identify the NF-YB transcription factor as a novel direct E2F1 target. Genome-wide expression analysis of the effects of NFYB knockdown on E2F1-mediated transcription identified a large group of genes that are co-regulated by E2F1 and NFYB. We also provide evidence that knockdown of NFYB enhances E2F1-induced apoptosis, suggesting a pro-survival function of the NFYB/E2F1 joint transcriptional program. Bioinformatic analysis suggests that deregulation of these NFY-dependent E2F1 target genes might play a role in sarcomagenesis as well as drug resistance.

## Introduction

E2Fs are a family of transcription factors important for the regulation of cell proliferation and apoptosis. E2F activation, resulting from cyclin-dependent kinase inhibition of retinoblastoma (Rb) protein function, is the trigger that leads to the transition from G_0_ to G_1_-S and initiation of the cell cycle. Genetic lesions in the Rb tumor suppressor pathway lead to unrestrained E2F activity and deregulated cell proliferation that are critical to the development of numerous cancers [1–3]. Among the three activator E2Fs, E2F1-E2F3, E2F1 is unique in its ability to induce apoptosis as well as proliferation [4]. Overexpression of E2F1 in quiescent fibroblasts induces apoptosis [5] while E2F1-/- mouse thymocytes are resistant to apoptotic stimuli [6]. The induction of numerous apoptotic genes and the repression of survival genes have been documented during E2F1-dependent apoptosis [7–12]. Additionally, DNA damage leads to E2F1 stabilization through ATM-mediated phosphorylation and activation through PCAF-mediated acetylation, resulting in apoptosis [7, 13]. Together, these studies suggest an important role of E2F1 deregulation in cancer as well as the therapeutic potential to harness the apoptotic activity of E2F1 for cancer therapy. However, better understanding of the control mechanisms balancing the proliferative and apoptotic activities of E2F1 is necessary to realize this potential.

We and others have previously demonstrated that E2F1 induces numerous other transcription factors, some of which cooperate with E2F1 during induction of target genes, such as the cooperation with FOXO1/3 to induce apoptotic genes [14]. In contrast, E2F1 induces TopBP1, which binds to E2F1 and specifically represses its apoptotic activity [15]. These examples underscore the importance of other transcriptional regulators in determining the transcriptional and phenotypic outcomes of E2F1.

The nuclear transcription factor Y (NF-Y) complex is a trimer that recognizes and binds to the CCAAT box, which shows a remarkable abundance in the promoters of genes regulated during the G_2_/M phase [16, 17]. NF-Y is composed of NFYA, NFYB, and NFYC subunits, all of which are necessary for DNA binding. NFYB and NFYC contain a histone-like motif and form a dimer which is required for association with NFYA and sequence-specific DNA binding [18]. The NF-Y complex plays a role in regulating proliferation by controlling expression of genes required for cell cycle progression such as cyclin A, cyclin B2, CDC25A, CDC25C, and CDK1 [19–22]. Furthermore, NF-Y regulates cell survival through direct control of several anti-apoptotic genes [23, 24]. Paradoxically, ectopic expression of NFYA was recently shown to induce apoptosis through upregulation of E2F1 [25].

Here we identify an inverse relationship, whereby E2F1 induces the expression of NFYB, which, together with E2F1, regulates a large group of joint target genes. Furthermore, we show that overexpression of these genes occurs in sarcomas compared to normal control tissues and associates with chemotherapy resistance. Taken together, our results identify NFYB as a new important pro-survival player in E2F1 transcriptional program.

## Materials and Methods

### Cell culture

U2OS human osteosarcoma cell line was obtained from Duke Cell Culture facility and its identity was authenticated by DNA STR profiling assay. Cells were grown in Dulbecco’s Modified Eagle Medium with 10% fetal bovine serum (FBS). 4-Hydroxytamoxifen (OHT) was obtained from Sigma (T176). Stable U2OS ER-E2F1 cell line was established by retroviral infection with pBabe-Puro-ER-HA-E2F1 (from K. Helin) followed by puromycin selection. For inducible knockdown of endogenous E2F1 shRNA targeting E2F1 was subcloned from pGIPZ-shE2F1 (Open Biosystems, V3LHS_393591) into the inducible pTRIPZ vector (Open Biosystems). U2OS cells were infected with lentiviral particles encoding pTRIPZ-E2F1 and stable line was established following puromycin selection. shRNA expression was induced by 1 μg/ml doxicyclin (Sigma).

### RNA isolation and RT-PCR

Total RNA was isolated using RNeasy Kit (Qiagen) on a QIAcube (Qiagen). RNA quality and concentration was assessed using Nanodrop ND-1000 Spectrophotometer. A total of 1μg RNA was reverse transcribed using High-Capacity cDNA Reverse Transcription Kit (Life Technologies 4368814) according to manufacturer’s instructions. cDNA was diluted 1:4 with water. cDNA was analyzed in triplicate by real time PCR on Life Technologies StepOnePlus Real-Time PCR Systems with Life Technologies Power SYBR Green Master Mix (4367659).

### Chromatin immunoprecipitation

ER-HA-E2F1 chromatin immunoprecipitations were conducted using the EZ-ChIP Kit from Millipore (17–371) according to the manufacturer’s instructions using monoclonal anti-HA antibody (Covance 16B12). NFYB (GPH1017391(-)01A), SFRP1 (GPH1026162 (+)01A), PRKCZ (GPH1000044(-)01A), FOLH1 (GPH1016343(-)01A), and IGX1A (GPH100001C(-)01A) EpiTect ChIP primers were obtained from Qiagen. Immunoprecipitated DNA was analyzed in quadruplicate by real time PCR on an ABI Prism 7900HT Sequence Detection System with RT^2^ SYBR Green ROX qPCR Mastermix (Qiagen 330520).

### Microarray analysis

RNA for microarray analysis of U2OS ER-HA-E2F1 cells was prepared using the RNeasy Kit (Qiagen) on a QIAcube (Qiagen). RNA quality and integrity was analyzed using Agilent Lab-on-a-Chip RNA Bioanalyzer. RNA was amplified using Ambion Message-Amp Premier Kit and analyzed on Affymetrix U133A 2.0 microarrays by the Duke Microarray Facility. Raw microarray data was normalized using the MAS5 algorithm. Expression data is available in the Gene Expression Omnibus database under accession number GSE61272.

### siRNA and lentivirus infection

siRNA reverse transfections were performed using Lipofectamine RNAiMax reagent (Life Technologies 13778150). Negative control siRNAs were from Sigma (SIC001) and Dharmacon (D-001810-01). siRNAs targeting NFYB were from Dharmacon (J-010002-08) and Ambion (4392420).

V5-tagged NFYB overexpression vector was obtained from DNASU (HsCD00329598). Lentiviral particles were produced using 293T cells co-transfected with the lentiviral vector, pMD2.G (Addgene 12259), and psPAX2 (Addgene 12260) using Mirus TransIT-LT1 Transfection Reagent (MIR 2300). U2OS ER-HA-E2F1 cells were infected followed by selection with blasticidin (10μg/ml) for two weeks.

### Western blotting

Cell pellets were lysed with RIPA buffer containing Complete Mini protease inhibitor cocktail (Roche) and PhosSTOP phosphatase inhibitor cocktail (Roche). Proteins were resolved on 10% SDS-PAGE, transferred to PFDF membranes and probed with antibodies against GAPDH (Santa Cruz, Sc-25778), NFYB (Santa Cruz, Sc-13045), cleaved PARP (Cell Signaling, 9541S), and V5 (Pierce, MA5-15253).

### Statistical analysis

Graphs and significance levels were generated using Graphpad software. Pooled results are presented as mean and standard deviation of triplicate experiments. For determining the list of genes regulated by NFYB, p values were calculated using two tailed t-test with unequal variance.

### Oncomine analysis

The list of genes generated from the microarray analysis (S1 Table) was entered into Oncomine Research Premium Edition as a custom concept and analyzed for associations across all Oncomine datasets with the following thresholds: p-value of 1E-4 and odds ratio of 2.

## Results

### NFYB is a direct target of E2F1

Previous work has documented the role of E2F transcription factors in the control of gene expression central to cell cycle and cell fate decisions [26]. Further work has provided evidence for a complex combinatorial mechanism of gene regulation involving E2F proteins together with other cooperating transcriptional regulators [27–29]. This includes regulatory cascades in which E2Fs activate the expression of genes encoding other transcription factors and then cooperate with these induced factors to regulate additional target genes, a relationship know as a feed-forward regulatory loop [14, 30]. To further explore this concept, with a focus on the E2F1 transcription factor, we have made use of an inducible system with an ER-E2F1 chimeric protein expressed in U2OS cells. Upon the addition of 4-hydroxytamoxifen (OHT), the chimeric protein translocates to the nucleus and activates E2F1-mediated transcription. These cells have been previously characterized showing minimal effect of OHT on the original parental cell line [14, 31].

In a recent study, we found that genes encoding transcription factors represent over 13% of E2F1 target genes with “Regulation of Transcription” as a significantly enriched Gene Ontology (GO) category (p<0.0001) [14]. Among the transcription factors induced by E2F1, we have focused on NFYB since NFY proteins, similar to E2F1, are important regulators of both proliferation and apoptosis (reviewed in [32]).

**Fig 1:**
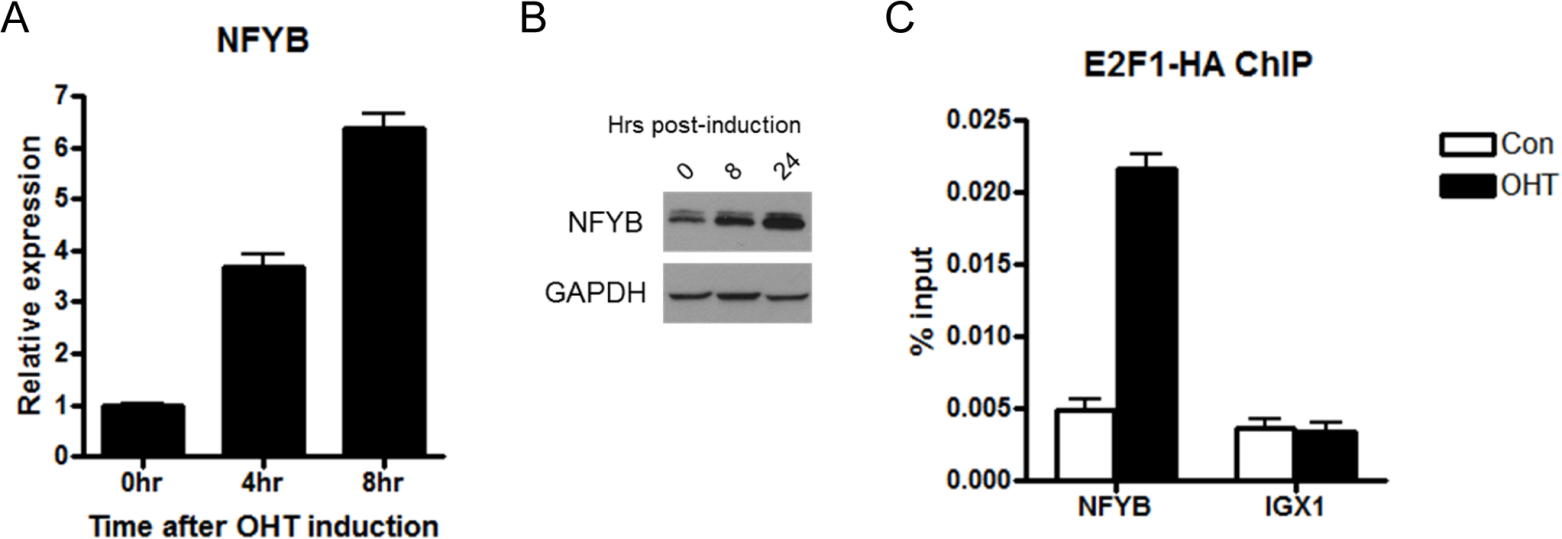
NFYB is a direct E2F1 target. (A) Real-time PCR analysis of NFYB mRNA levels at four and eight hours after E2F1 induction in U2OS ER-E2F1 cells. Cells were serum starved for twenty four hours prior to addition of 80 nM OHT. (B) Western blot of NFYB and GAPDH protein levels. U2OS ER-E2F1 cells were induced with 80nM OHT for eight and twenty four hours following twenty four hour serum starvation. Lysates were analyzed by SDS-PAGE/Western blot and probed with anti-NFYB and anti-GAPDH antibodies (loading control). (C) U2OS ER-E2F1 cells were serum starved for twenty four hours followed by induction with 80nM OHT for seven hours. Chromatin immunoprecipitation was performed with anti-HA antibody for detection of ER-HA E2F1 binding to the NFYB promoter and IGX1 repeats (negative control).

To validate the microarray results showing induction of NFYB by E2F1, we analyzed NFYB expression levels by real-time PCR. Following OHT induction, NFYB expression increased linearly through four and eight hours to reach a level almost seven-fold higher than prior to E2F1 induction (Fig 1A). This induction of RNA is also reflected in protein level by Western blotting where NFYB induction can be seen at eight and twenty four hours following OHT induction (Fig 1B).

Next, we examined the role of endogenous E2F activity in NFYB regulation. We found that the knockdown of E2F1 in U2OS cells resulted in small but statistically significant downregulation of NFYB mRNA levels (A in S1 Fig). In a complementary approach, we made use of a publicly available microarray expression dataset (GEO: GSE16454) of intestinal villi from tissue specific conditional Rb knockout mice [33]. Since Rb is a negative upstream regulator of E2Fs, inactivation of Rb is expected to result in the upregulation of endogenous E2F target genes [34]. As demonstrated in B in S1 Fig, NFYB expression was significantly higher in Rb knockout compared to wild-type villi to support the concept that E2F activation upregulates NFYB.

To address whether NFYB induction is a direct effect of the action of E2F1, we performed a chromatin-immunoprecipitation (ChIP) analysis to determine whether E2F1 binds to the NFYB promoter. Following induction of E2F1 activity in our U2OS ER-E2F1 system by OHT, E2F1 binding to the NFYB promoter increased more than four-fold while binding to IGX1, a negative control, did not change (Fig 1C). To further extend these findings to an additional cell type and to evaluate the binding of endogenous E2F1 to the NFYB promoter, we analyzed ChIP-Seq data from the ENCODE project [35]. We found that endogenous E2F1 in HeLa cells bound to the promoter of NFYB in a region near the transcriptional start site (A in S2 Fig). The degree of E2F1 occupancy in the NFYB promoter was comparable to those of known E2F1 targets APAF1, Cyclin E, and E2F1 (B in S2 Fig).

Taken together, our results demonstrate that NFYB is a novel direct E2F1 target.

### Role of NFYB in E2F1-mediated transcriptional activation

**Fig 2:**
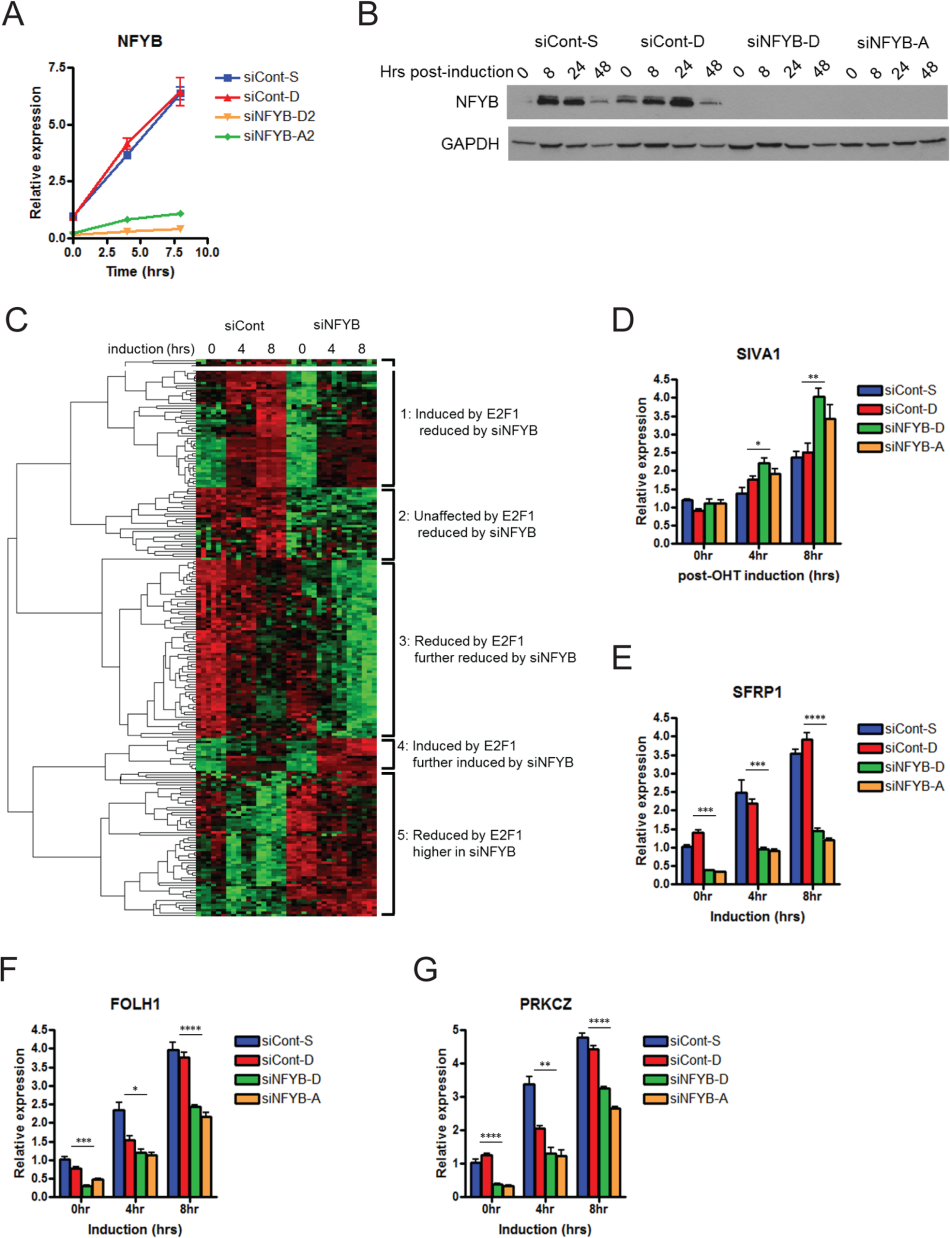
Identification of NFYB-dependent E2F1 target genes. (A) Real-time PCR analysis of NFYB mRNA levels following siRNA transfection. U2OS ER-E2F1 cells were transfected with two siRNAs targeting NFYB or two negative control siRNAs at 100nM. Cells were serum starved for twenty four hours followed by 80nM OHT induction for four or eight hours. (B) Western blot analysis of NFYB protein levels following E2F1 activation. U2OS ER-E2F1 cells transfected with siRNA targeting NFYB or control were serum starved for twenty four hours followed by OHT induction at eight, twenty four, and forty eight hours. Lysates were analyzed by SDS-PAGE/Western blot and probed with anti-NFYB and anti-GAPDH antibodies (loading control). (C) Microarray analysis of the effect of NFYB knockdown on E2F1-mediated transcription. Samples were processed in the same manner as in Fig 2A and analyzed using Human U133A 2.0 expression microarrays. Heatmap represents the results of hierarchical clustering of 224 probes that showed at least 1.3-fold increase or 0.7-fold decrease in expression of compared to control following NFYB knockdown at eight hours. Real-time PCR validation of target gene expression decrease for SIVA1 (D), SFRP1 (E), FOLH1 (F), and PRKCZ (G) following NFYB knockdown. Samples were processed in the same manner as Fig 2A. * denotes p<0.05, ** denotes p<0.01, *** denotes p<0.001, **** denotes p<0.0001.

To explore the role of NFYB in E2F1-mediated transcriptional responses, we knocked down the expression of NFYB using two different small interfering RNA (siRNAs) targeting NFYB. Efficient knockdown of NFYB mRNA levels was validated by real-time PCR (Fig 2A) and Western blot analysis (Fig 2B).

We next interrogated the genome-wide effects of NFYB knockdown on E2F1-mediated transcription using expression microarrays (deposited into GEO database as GSE61272). Comparison of genome-wide expression levels after E2F1 activation in cells transfected with either negative control siRNAs or siRNAs targeting NFYB identified 197 genes whose expression in E2F1 activated cells (after 8 hours of OHT induction) is significantly different (p<0.001) following NFYB knockdown by at least 1.3 fold higher or 0.7 fold lower compared to control siRNA. As expression of these genes is affected by NFYB knockdown and given that E2F1 directly targets NFYB, these genes represent the broad spectrum of genes directly or indirectly targeted by E2F1 and NFYB. Unsupervised clustering of these genes reveals five clusters of genes: 1) induced by E2F1 activation and reduced by NFYB knockdown, 2) unaffected by E2F1 activation and reduced by NFYB knockdown, 3) reduced by E2F1 activation and further reduced by NFYB knockdown, 4) induced by E2F1 activation and further induced by NFYB knockdown, 5) reduced by E2F1 but higher in NFYB knockdown (Fig 2C and S1 Table).

Further examination of the genes within the clusters revealed multiple genes involved in apoptosis and survival. For example, within cluster 2 (expression reduced by NFYB knockdown), the levels of API5, an inhibitor of E2F1-mediated apoptosis, and of MALT1, a paracaspase that promotes activation of NF-κB signaling was reduced following NFYB knockdown [36, 37]. Within cluster 4 (genes induced by E2F1 and further increased by NFYB knockdown) is SIVA1, apoptosis inducing factor, a proapoptotic gene that plays an important role in CD27-mediated apoptosis [38] and is known to be induced by E2F1 [39]. Real time RT-PCR validation of these results is shown for SIVA1 (Fig 2D) as well as API5 (S3 Fig).

Focused analysis of the genes positively regulated by both E2F1 and NFYB identified 148 genes whose expression is induced at least two fold by E2F1 activation and significantly lower (p<0.05) following NFYB knockdown at a ratio of 1:1.2 compared to control (S2 Table). To further validate these genes that may be co-regulated by E2F1 and NFYB, we used real-time RT-PCR to measure the expression of those genes in which the effect of NFYB knockdown on expression levels was most significant. Validation for selected genes is presented for SFRP1 (Fig 2E), FOLH1 (Fig 2F), and PRKCZ (Fig 2G).

### NFYB-dependent E2F1 target genes

**Fig 3:**
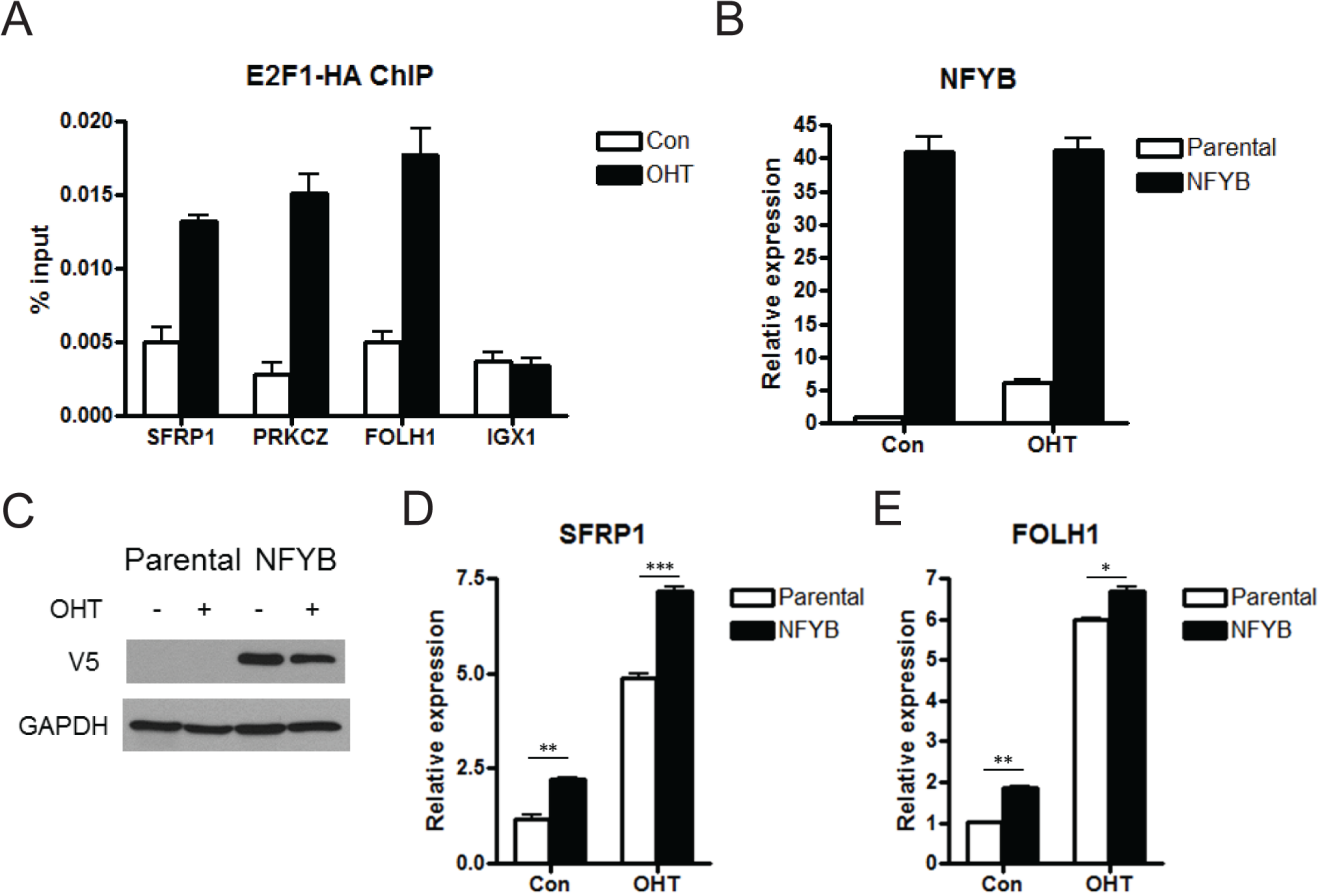
NFYB-dependent E2F1 targets. (A) ChIP analysis of E2F1 binding to target gene promoters. U2OS ER-HA E2F1 cells were serum starved for twenty four hours followed by 80nM OHT induction for seven hours. Immunoprecipitation was performed with anti-HA antibody for detection of ER-HA E2F1 binding to the SFRP1, PRKCZ, FOLH1 promoters and to IGX1 repeats (negative control). (B) Real-time PCR analysis of NFYB expression. U2OS ER-E2F1 cells were infected with lentviruses encoding the NFYB cDNA tagged with V5. A stable line was selected using blasticidin resistance for two weeks. This NFYB overexpression line is denoted as NFYB. The parental U2OS ER-E2F1 line is denoted as “parental”. Cell lines were serum starved for twenty four hours followed by 80nM OHT induction for eight hours. (C) Western blot analysis of ectopic NFYB expression following establishment of stable NFYB overexpression cell line. Lysates were analyzed by western blotting of NFYB transgene levels using an anti-V5 antibody, and GAPDH. Samples were processed in the same manner as in Fig 3B. Real-time PCR analysis of SFRP1 (D) and FOLH1 (E) in U2OS ER-E2F1 in parental and NFYB overexpressing U2OS ER-E2F1 cell lines. Samples were processed in the same manner as in Fig 3B.

To establish whether these genes induced by E2F1 in an NFYB dependent manner are direct targets of E2F1, we performed chromatin immunoprecipitation (ChIP) analysis before and after induction by OHT. All three genes showed increased E2F1 binding to their promoters following E2F1 induction (Fig 3A). E2F1 recruitment specificity was demonstrated by the lack of E2F1 binding to the IGX1 negative control sequences. These results indicate that E2F1 is physically associated with the regulatory regions of these genes to regulate their expression. As a complementary approach to further validate the involvement of NFYB in induction of these targets, we employed a gain of function approach. To this end, we infected the inducible U2OS ER-E2F1 cell line with a lentivirus containing NFYB cDNA. Following antibiotic selection to establish a stable line, we validated NFYB ectopic expression by RT-PCR (Fig 3B) and Western blotting (Fig 3C).

To test whether increased expression of NFYB caused induction of the target genes, we induced the transgenic and parental line with OHT and compared target gene expression. Real-time PCR analysis demonstrated that overexpression of NFYB alone induced SFRP1 (Fig 3D) and FOLH1 (Fig 3E). Expression of these genes was also higher in NFYB-overexpressing cells following E2F1 induction. Taken together with the siRNA data, these results demonstrate that SFRP1 and FOLH1 are novel targets that are co-regulated by E2F1 and NFYB.

**Fig 4:**
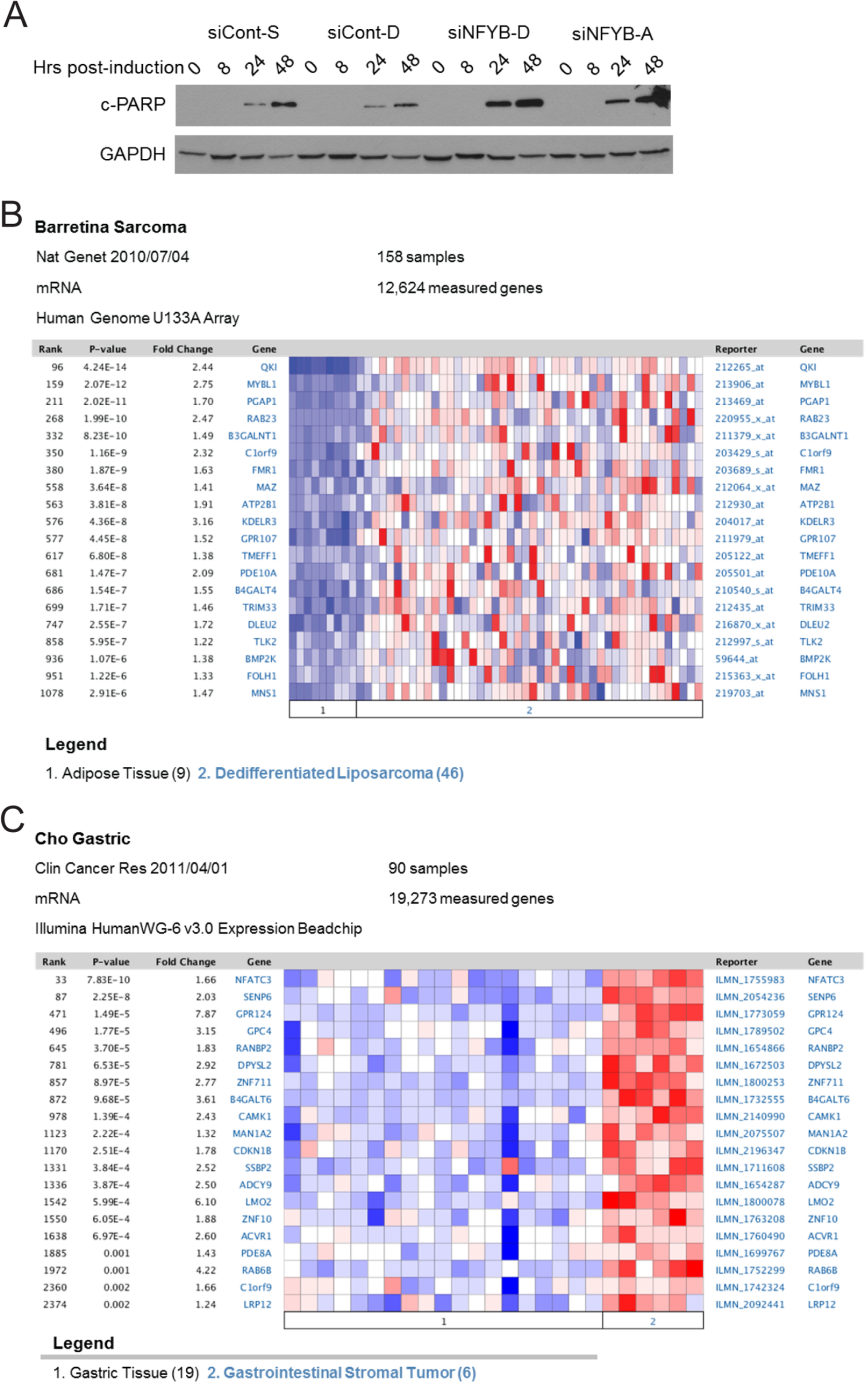
NFYB signature overexpression associates with sarcoma. (A) Western blot analysis of cleaved PARP levels following E2F1 activation. U2OS ER-E2F1 cells transfected with siRNA targeting NFYB or control were serum starved for twenty four hours followed by OHT induction at eight, twenty four, and forty eight hours. Lysates were analyzed by SDS-PAGE/Western blot and probed with anti-cleaved PARP and anti-GAPDH antibodies (loading control). See Fig 2B for NFYB knockdown efficiency in these samples. (B) Oncomine analysis demonstrating upregulation of genes from the NFYB-dependent E2F1 signature in Barretina sarocoma dataset. Heatmap showing higher expression in dedifferentiated liposarcoma compared to normal adipose tissue. Red color designates high expression and blue color designates low expression. (C) Oncomine analysis demonstrating upregulation of genes from the NFYB-dependent E2F1 signature in gastrointestinal stromal tumor compared to normal gastric tissue.

Given the effect of NFYB on the expression of genes involved in apoptosis, we sought to determine whether NFYB induction played a functional role in E2F1-mediated apoptosis. Western blot analysis following knockdown of NFYB by two different siRNAs demonstrated significantly increased E2F1-induced apoptosis as evidenced by the increased intensity of cleaved PARP protein band (Fig 4A and Fig 2B demonstrating knockdown efficiency). Coupled with our microarray data that identified multiple pro-survival NFYB target genes, this functional result suggests that induction of NFYB by E2F1 contributes to the attenuation of the apoptotic arm of E2F1 signaling.

### Genes from an E2F1-NFYB signature are upregulated in sarcoma and drug resistant cell lines

In light of the established roles of E2F and NFY in cancer phenotypes, we sought to determine whether the transcriptional program jointly controlled by E2F1 and NFYB might define unique cancer states. Using the results from the microarray analysis, we utilized the 174 expression probes (148 genes) generated earlier (S2 Table) as an E2F1/NFYB signature. We made use of the Oncomine analysis suite, which integrates genome-wide expression and copy number data from more than 700 cancer-related datasets as a rich source of expression data coupled with phenotypic information [40]. An Oncomine query with the E2F1/NFYB signature revealed sarcoma as the most significant cancer type in tumor versus normal analysis (A in S1 Fig). In five independent sarcoma datasets, the E2F1/NFYB signature is significantly associated with signatures (concepts) consisting of genes that are overexpressed in cancer compared to normal tissue. A representative heatmap shows the overexpression of the genes in the E2F1/NFY signature in dedifferentiated liposarcoma compared to normal adipose tissue (Fig 4B). A second representative heatmap shows the overexpression of E2F1/NFY signature genes in gastrointestinal stromal tumors compared to normal gastric tissue (Fig 4C).

To determine whether this association is specific for the E2F1/NFYB signature, we compared the results to those signatures generated using the Oncomine determined concept “Up-regulated genes in human osteosarcoma cell line (U2OS) expressing E2F1 compared to U2OS controls – Literature-defined Concepts” (E2F1 signature). This signature did not show the same enrichment within sarcoma datasets, with only one dataset in which the signature significantly associates with a concept overexpressed in cancer compared to normal (A in S4 Fig). Similarly, an NFYB signature in which the top 145 genes that were most strongly downregulated by NFYB knockdown irrespective of E2F1 showed only one dataset in which the signature significantly associates with a concept overexpressed in cancer and one in which it associates with a concept underexpressed in cancer. Together with the data showing that NFYB is protective against E2F1-mediated apoptosis, these results suggest that the NFYB-dependent E2F1 transcriptional program we defined may be involved in the oncogenesis or maintenance of sarcomas.

**Fig 5:**
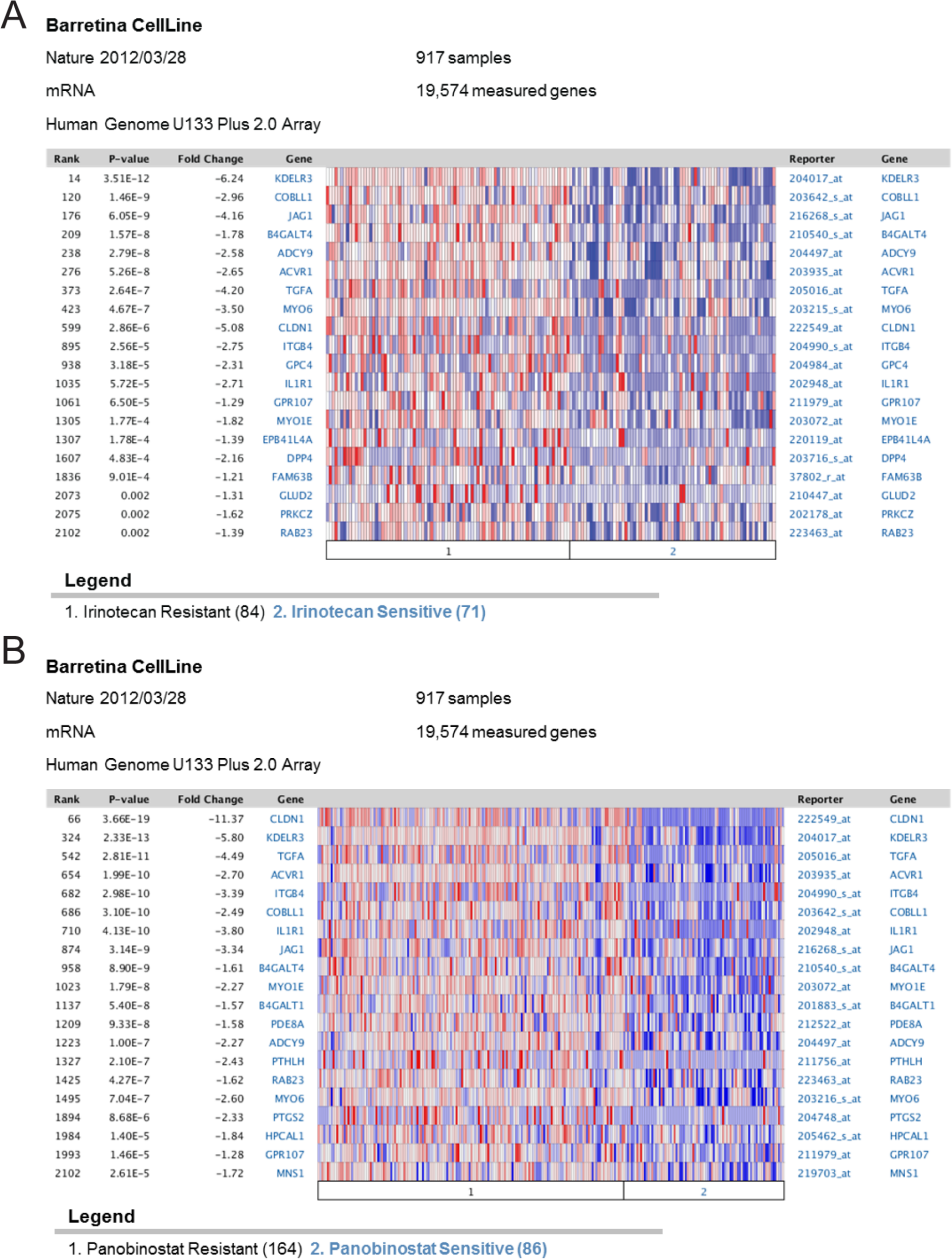
NFYB signature overexpression associates with drug therapy resistance. (A) Oncomine analysis demonstrating upregulation of genes from the NFYB-dependent E2F1 signature in in irinotecan resistant cell lines compared to irinotecan sensitive cell lines. (B) Oncomine analysis demonstrating upregulation of genes from the NFYB-dependent E2F1 signature in panobinostat resistant cell lines compared to panobinostat sensitive cell lines.

The Oncomine analysis further indicated lower expression of the E2F1/NFYB signature in drug sensitive sarcoma cell lines compared with drug resistant lines in six independent datasets (B in S4 Fig). This association is not limited to sarcoma, but persists across a broad range of cell lines. Two example heatmaps show overexpression of signature genes in irinotecan resistant cell lines (Fig 5A) and panobinostat resistant cell lines (Fig 5B) compared to sensitive cell lines. We again explored whether this association was specific to the joint E2F1/NFYB signature by comparing it to similar analysis with the E2F1 signature and NFYB signature. Unlike the joint signature, the separate E2F1 and NFYB signatures did not show a clear association with drug resistance (S4 Fig).

In summary, when considered together with data showing NFYB as protective against E2F1-mediated apoptosis, this analysis suggests that the joint E2F1/NFYB transcriptional program may play a role in cancer resistance to chemotherapy.

## Discussion

E2Fs play a critical role in many cell functions including proliferation, development, and cell death. Among the E2Fs, E2F1 is of particular interest because it activates expression of genes involved in both proliferation and apoptosis [41], with studies detailing different transcription factor partners to determine specificity towards one or the other [14]. Our study identifies NFYB as a novel direct E2F1 target that, in turn, regulates multiple downstream genes.

Both E2F1 and NF-Y are recognized as important regulators of cell cycle progression, where E2F1 is mostly known for its key role in G1/S progression and NF-Y in G2/M phase. Several previous studies reported examples where E2F1 and NF-Y proteins were shown to bind to the promoters of the same genes [42, 43]. Furthermore, genome-wide analyses identified enrichment for both NF-Y and E2F binding sites in a large cluster of genes upregulated following p53 inactivation in an in-vitro transformation system. This cluster is highly enriched for cell cycle genes and its upregulation is associated with poor prognosis in multiple cancer types [44]. Here we demonstrate that NFYB is a direct transcriptional target of E2F1, suggesting that joint gene regulation by E2F1 and NF-Y might not be independent but rather represent an example of a feed-forward loop. One role of such regulatory network motif might be in coupling the G1/S transition where E2F activity is maximal with the G2/M phase of cell cycle where both E2F and NFY activities are important. In this context, the anti-apoptotic activity of the joint E2F1/NFYB transcriptional program that we describe here might be important to counteract the intrinsic pro-apoptotic E2F1 activity during cell proliferation.

The regulomes of E2F1 and NFYB partially overlap and in the current study we focused on a group of genes that are positively regulated by both transcription factors. Based on previously published studies we suggest that some of these joint targets are responsible for the attenuation of E2F1-induced apoptosis [36, 37], thus suggesting a complex interplay regulating the final apoptotic phenotype. Furthermore, in silico analysis of this subset of co-regulated genes suggests that the NFYB-dependent E2F1 program is upregulated and linked with various types of sarcoma when compared to corresponding normal tissue. This analysis furthermore demonstrates that E2F1/NFYB signature is associated with chemotherapy resistance. These in vivo associations are consistent with the pro-survival function of NFYB as noted in the increased apoptosis caused by the silencing of NFYB.

In addition to genes induced by both E2F1 and NFYB, our clustering analysis also identified a group of genes that are reduced by E2F1 in an NFYB-dependent manner (Fig 2B, cluster 5). These include multiple mitotic genes, such as CDC25C as well as the other two subunits of the NFY complex, NFYA and NFYC. These results may indicate a compensatory induction of NFYA and NFYC in response to the depletion of NFYB. Additional studies are needed to examine the functional significance of this complex interplay between E2F1 and different subunits of the NFY complex in cell cycle progression and other biological processes.

A recent study has shown that E2F1 is transcriptionally induced by ectopic NFYA expression and that NFYA binds to the E2F1 promoter [25]. Combined with our results showing that E2F1 induces NFYB by direct binding to its promoter indicates a positive feedback loop in which the two transcription factors amplify one another’s expression. Notably, however, whereas NFYA was shown to promote apoptosis by inducing E2F1, our results suggest a pro-survival function for NFYB. Such opposing effects of the two subunits of the NF-Y complex might reveal different aspects of co-regulation and feedback mechanisms between E2F1 and NF-Y or be a result of different approaches employed (ectopic expression of NFYA in Gurtner et al vs knockdown of the endogenous NFYB in our study) or different cell lines used.

Our study identified several pro and anti-apoptotic genes regulated by NF-Y and E2F1. API5, an apoptosis inhibitor, is down-regulated while SIVA1, an apoptosis-inducing factor, is upregulated following NFYB knockdown. This result matches phenotypically with increased E2F1-mediated apoptosis upon NFYB depletion. Further studies are necessary to determine the roles of specific pro and anti-apoptotic targets regulated by NFYB and E2F1 in E2F1-mediated apoptosis.

Finally, our cancer vs normal analysis using a set of genes jointly induced by E2F1 and NFYB demonstrated most significant overexpression in sarcoma. This is intriguing in that the inducible E2F1 system used in this study comes from an osteosarcoma, indicating that the NFYB-dependent E2F1 transcriptional program defined here might be tissue-specific. Further enhancing this idea is the fact that E2F1 target genes induced in an NFYB-independent manner do not show this association with sarcoma. Our results showing NFYB to be protective against E2F1 mediated apoptosis moreover suggest this NFYB/E2F1 regulated gene set may be involved in the development or survival of sarcoma cells. We also found that a subset of NFYB-dependent E2F1 regulated genes is overexpressed in chemotherapy resistant cell lines. This is not limited to a narrow range of chemotherapies, as the association is found for both a topoisomerase inhibitor (irinotecan) and an HDAC inhibitor (panobinostat). Importantly, this association is not limited to sarcoma as it is observed when comparing resistant vs sensitive cell lines from multiple tissue types. Accordingly, potential use of the NFYB/E2F signature can be explored as a biomarker for selecting patients that may respond to certain chemotherapies in future clinical trials.

## Supporting Information

S1 Fig Effect of endogenous E2F activity on NFYB expression. Real time qPCR analysis of E2F1 and NFYB expression following 24 hours doxycyclin-inducible E2F1 knockdown in U2OS cells (A). Publicly available microarray expression data of the intestinal villi from wild type and RB knockout mouse (GSE16454) was analyzed for the expression of NFYB. Data presented are means +/- SD of triplicate samples (reversed logged RMA data for probeset 1419266_at). Asterisks denote statistically significant difference between the two groups as evaluated by t-test (*p<0.05, **p<0.01) (B).
(TIF)

S2 Fig Analysis of ENCODE ChIP-seq data for binding of endogenous E2F1 to NFYB promoter. A screen shot of UCSC Genome Browser showing NFYB promoter. Tracks on the bottom represent ChIP-seq signals for different indicated transcription factors with the intensity of black color proportional to the signal strength. Strong binding signal of endogenous E2F1 to the region around the NFYB transcription start site in HeLa cells is boxed (A). The signals of normalized bound reads for E2F1 ChIP-seq in HeLa cells for NFYB, CCNE1, APAF1 and E2F1 promoters were extracted using UCSC Genome Browser and plotted (B).
(TIF)

S3 Fig API5 expression decreases following NFYB knockdown. Real-time PCR validation of target gene expression decrease for API5. Samples were processed in the same manner as Fig 2A. * denotes p<0.05, ** denotes p<0.01.
(TIF)

S4 Fig Overview of Oncomine analysis. Number of sarcoma datasets from cancer vs normal Oncomine analysis in which signatures (concepts) consisting of differentially expressed genes are significantly associated with the indicated signatures. The NFYB-dependent E2F1 signature (Fig 2D and S2 Table) is derived from our microarray analysis and described in the Results section. The E2F1 (U2OS induced) signature is a concept defined by Oncomine based on previously published microarray analysis of genes upregulated in U2OS ER-E2F1 cells following OHT induction. The E2F1-independent NFYB signature consists of probes (174) which expression is reduced 30% following NFYB knockdown regardless of the effect of E2F1 induction. Significance is set at an odds ratio of at least two and p value of less than 0.0001. Red indicates number of signatures overexpressed in cancer compared to normal cells. Blue represents number of signatures underexpressed in cancer compared to normal (A). Number of datasets in which concepts from therapy sensitivity datasets are significantly associated with the signatures described in (A in S4 Fig). Significance is set at an odds ratio of at least two and p value of less than 0.0001. Red indicates number of signatures overexpressed in drug resistant compared to drug sensitive cells. Blue represents number of signatures underexpressed in resistant compared to sensitive cells (B).
(TIF)

S1 Table Genes groups from clustering of genes affected by NFYB downregulation. Unsupervised hierarchical clustering was performed on genes whose expression is significantly different (p<0.001) in E2F1 activated cells following NFYB knockdown by at least 1.3 fold higher or 0.7 fold lower compared to control siRNA, resulting in 5 groups: 1) induced by E2F1 activation and reduced by NFYB knockdown, 2) unaffected by E2F1 activation and reduced by NFYB knockdown, 3) reduced by E2F1 activation and further reduced by NFYB knockdown, 4) induced by E2F1 activation and further induced by NFYB knockdown, 5) reduced by E2F1 but higher in NFYB knockdown.
(PDF)

S2 Table Genes induced by E2F1 activation and downregulated following NFYB knockdown. Analysis was performed on microarray data showing 148 genes whose expression is induced at least two fold by E2F1 activation and significantly lower (p<0.05) following NFYB knockdown at a ratio of 1:1.2 compared to control.
(PDF)

## References

[1] Knudsen ES, Knudsen KE. Retinoblastoma tumor suppressor: where cancer meets the cell cycle. Exp Biol Med (Maywood). 2006;231(7):1271–81. . 16816134 10.1177/153537020623100713

[2] Bosco EE, Knudsen ES. RB in breast cancer: at the crossroads of tumorigenesis and treatment. Cell Cycle. 2007;6(6):667–71. . 17361100 10.4161/cc.6.6.3988

[3] Johnson DG, Degregori J. Putting the Oncogenic and Tumor Suppressive Activities of E2F into Context. Curr Mol Med. 2006;6(7):731–8. . 17100599 10.2174/1566524010606070731

[4] Ginsberg D. E2F1 pathways to apoptosis. FEBS Lett. 2002;529(1):122–5. . 12354623 10.1016/s0014-5793(02)03270-2

[5] Kowalik TF, DeGregori J, Schwarz JK, Nevins JR. E2F1 overexpression in quiescent fibroblasts leads to induction of cellular DNA synthesis and apoptosis. J Virol. 1995;69(4):2491–500. 7884898 10.1128/jvi.69.4.2491-2500.1995 PMC188925

[6] Field SJ, Tsai FY, Kuo F, Zubiaga AM, Kaelin WG Jr., Livingston DM, et al E2F-1 functions in mice to promote apoptosis and suppress proliferation. Cell. 1996;85(4):549–61. . 8653790 10.1016/s0092-8674(00)81255-6

[7] Pediconi N, Ianari A, Costanzo A, Belloni L, Gallo R, Cimino L, et al Differential regulation of E2F1 apoptotic target genes in response to DNA damage. Nat Cell Biol. 2003;5(6):552–8. 10.1038/ncb998 . 12766778

[8] Irwin M, Marin MC, Phillips AC, Seelan RS, Smith DI, Liu W, et al Role for the p53 homologue p73 in E2F-1-induced apoptosis. Nature. 2000;407(6804):645–8. 10.1038/35036614 . 11034215

[9] Nahle Z, Polakoff J, Davuluri RV, McCurrach ME, Jacobson MD, Narita M, et al Direct coupling of the cell cycle and cell death machinery by E2F. Nat Cell Biol. 2002;4(11):859–64. 10.1038/ncb868 . 12389032

[10] Hershko T, Ginsberg D. Up-regulation of Bcl-2 homology 3 (BH3)-only proteins by E2F1 mediates apoptosis. J Biol Chem. 2004;279(10):8627–34. 10.1074/jbc.M312866200 . 14684737

[11] Croxton R, Ma Y, Song L, Haura EB, Cress WD. Direct repression of the Mcl-1 promoter by E2F1. Oncogene. 2002;21(9):1359–69. 10.1038/sj.onc.1205157 . 11857079

[12] Cassimere EK, Pyndiah S, Sakamuro D. The c-MYC-interacting proapoptotic tumor suppressor BIN1 is a transcriptional target for E2F1 in response to DNA damage. Cell Death Differ. 2009;16(12):1641–53. 10.1038/cdd.2009.98 . 19629135

[13] Lin WC, Lin FT, Nevins JR. Selective induction of E2F1 in response to DNA damage, mediated by ATM-dependent phosphorylation. Genes Dev. 2001;15(14):1833–44. 11459832 PMC312742

[14] Shats I, Gatza ML, Liu B, Angus SP, You L, Nevins JR. FOXO transcription factors control E2F1 transcriptional specificity and apoptotic function. Cancer Res. 2013;73(19):6056–67. 10.1158/0008-5472.CAN-13-0453 23966291 PMC3815650

[15] Liu K, Luo Y, Lin FT, Lin WC. TopBP1 recruits Brg1/Brm to repress E2F1-induced apoptosis, a novel pRb-independent and E2F1-specific control for cell survival. Genes Dev. 2004;18(6):673–86. Epub 2004/04/13. doi: 10.1101/gad.1180204 18/6/673 [pii]. 15075294 10.1101/gad.1180204 PMC387242

[16] Elkon R, Linhart C, Sharan R, Shamir R, Shiloh Y. Genome-wide in silico identification of transcriptional regulators controlling the cell cycle in human cells. Genome Res. 2003;13(5):773–80. 10.1101/gr.947203 12727897 PMC430898

[17] Imbriano C, Gurtner A, Cocchiarella F, Di Agostino S, Basile V, Gostissa M, et al Direct p53 transcriptional repression: in vivo analysis of CCAAT-containing G2/M promoters. Mol Cell Biol. 2005;25(9):3737–51. 10.1128/MCB.25.9.3737-3751.2005 15831478 PMC1084283

[18] Romier C, Cocchiarella F, Mantovani R, Moras D. The NF-YB/NF-YC structure gives insight into DNA binding and transcription regulation by CCAAT factor NF-Y. J Biol Chem. 2003;278(2):1336–45. 10.1074/jbc.M209635200 . 12401788

[19] Bolognese F, Wasner M, Dohna CL, Gurtner A, Ronchi A, Muller H, et al The cyclin B2 promoter depends on NF-Y, a trimer whose CCAAT-binding activity is cell-cycle regulated. Oncogene. 1999;18(10):1845–53. 10.1038/sj.onc.1202494 . 10086339

[20] Farina A, Manni I, Fontemaggi G, Tiainen M, Cenciarelli C, Bellorini M, et al Down-regulation of cyclin B1 gene transcription in terminally differentiated skeletal muscle cells is associated with loss of functional CCAAT-binding NF-Y complex. Oncogene. 1999;18(18):2818–27. 10.1038/sj.onc.1202472 . 10362252

[21] Korner K, Jerome V, Schmidt T, Muller R. Cell cycle regulation of the murine cdc25B promoter: essential role for nuclear factor-Y and a proximal repressor element. J Biol Chem. 2001;276(13):9662–9. 10.1074/jbc.M008696200 . 11104768

[22] Zwicker J, Lucibello FC, Wolfraim LA, Gross C, Truss M, Engeland K, et al Cell cycle regulation of the cyclin A, cdc25C and cdc2 genes is based on a common mechanism of transcriptional repression. EMBO J. 1995;14(18):4514–22. 7556094 10.1002/j.1460-2075.1995.tb00130.x PMC394543

[23] Dolfini D, Zambelli F, Pavesi G, Mantovani R. A perspective of promoter architecture from the CCAAT box. Cell Cycle. 2009;8(24):4127–37. . 19946211 10.4161/cc.8.24.10240

[24] Benatti P, Basile V, Merico D, Fantoni LI, Tagliafico E, Imbriano C. A balance between NF-Y and p53 governs the pro- and anti-apoptotic transcriptional response. Nucleic Acids Res. 2008;36(5):1415–28. 10.1093/nar/gkm1046 18187512 PMC2275158

[25] Gurtner A, Fuschi P, Martelli F, Manni I, Artuso S, Simonte G, et al Transcription factor NF-Y induces apoptosis in cells expressing wild-type p53 through E2F1 upregulation and p53 activation. Cancer Res. 2010;70(23):9711–20. 10.1158/0008-5472.CAN-10-0721 . 20952509

[26] Polager S, Ginsberg D. p53 and E2f: partners in life and death. Nat Rev Cancer. 2009;9(10):738–48. Epub 2009/09/25. doi: 10.1038/nrc2718 nrc2718 [pii]. . 19776743 10.1038/nrc2718

[27] Giangrande PH, Hallstrom TC, Tunyaplin C, Calame K, Nevins JR. Identification of E-box factor TFE3 as a functional partner for the E2F3 transcription factor. Mol Cell Biol. 2003;23(11):3707–20. Epub 2003/05/16. . 12748276 10.1128/MCB.23.11.3707-3720.2003 PMC155231

[28] Hallstrom TC, Nevins JR. Jab1 is a specificity factor for E2F1-induced apoptosis. Genes Dev. 2006;20(5):613–23. Epub 2006/02/17. doi: gad.1345006 [pii] 10.1101/gad.1345006 . 16481464 PMC1410801

[29] Schlisio S, Halperin T, Vidal M, Nevins JR. Interaction of YY1 with E2Fs, mediated by RYBP, provides a mechanism for specificity of E2F function. EMBO J. 2002;21(21):5775–86. 12411495 10.1093/emboj/cdf577 PMC131074

[30] Lu H, Hallstrom TC. The nuclear protein UHRF2 is a direct target of the transcription factor E2F1 in the induction of apoptosis. J Biol Chem. 2013;288(33):23833–43. 10.1074/jbc.M112.447276 23833190 PMC3745330

[31] Muller H, Bracken AP, Vernell R, Moroni MC, Christians F, Grassilli E, et al E2Fs regulate the expression of genes involved in differentiation, development, proliferation, and apoptosis. Genes Dev. 2001;15(3):267–85. 10.1101/gad.864201 11159908 PMC312619

[32] Gatta R, Dolfini D, Mantovani R. NF-Y joins E2Fs, p53 and other stress transcription factors at the apoptosis table. Cell Death Dis. 2011;2:e162 10.1038/cddis.2011.45 21614092 PMC3122124

[33] Chong JL, Wenzel PL, Saenz-Robles MT, Nair V, Ferrey A, Hagan JP, et al E2f1-3 switch from activators in progenitor cells to repressors in differentiating cells. Nature. 2009;462(7275):930–4. 10.1038/nature08677 20016602 PMC2806193

[34] Chellappan SP, Hiebert S, Mudryj M, Horowitz JM, Nevins JR. The E2F transcription factor is a cellular target for the RB protein. Cell. 1991;65(6):1053–61. . 1828392 10.1016/0092-8674(91)90557-f

[35] Consortium EP. An integrated encyclopedia of DNA elements in the human genome. Nature. 2012;489(7414):57–74. 10.1038/nature11247 22955616 PMC3439153

[36] Morris EJ, Michaud WA, Ji JY, Moon NS, Rocco JW, Dyson NJ. Functional identification of Api5 as a suppressor of E2F-dependent apoptosis in vivo. PLoS Genet. 2006;2(11):e196 10.1371/journal.pgen.0020196 17112319 PMC1636698

[37] Qiao Q, Yang C, Zheng C, Fontan L, David L, Yu X, et al Structural architecture of the CARMA1/Bcl10/MALT1 signalosome: nucleation-induced filamentous assembly. Mol Cell. 2013;51(6):766–79. 10.1016/j.molcel.2013.08.032 24074955 PMC3929958

[38] Prasad KV, Ao Z, Yoon Y, Wu MX, Rizk M, Jacquot S, et al CD27, a member of the tumor necrosis factor receptor family, induces apoptosis and binds to Siva, a proapoptotic protein. Proc Natl Acad Sci U S A. 1997;94(12):6346–51. 9177220 10.1073/pnas.94.12.6346 PMC21052

[39] Fortin A, MacLaurin JG, Arbour N, Cregan SP, Kushwaha N, Callaghan SM, et al The proapoptotic gene SIVA is a direct transcriptional target for the tumor suppressors p53 and E2F1. J Biol Chem. 2004;279(27):28706–14. 10.1074/jbc.M400376200 . 15105421

[40] Rhodes DR, Kalyana-Sundaram S, Mahavisno V, Varambally R, Yu J, Briggs BB, et al Oncomine 3.0: genes, pathways, and networks in a collection of 18,000 cancer gene expression profiles. Neoplasia. 2007;9(2):166–80. 17356713 10.1593/neo.07112 PMC1813932

[41] DeGregori J, Johnson DG. Distinct and Overlapping Roles for E2F Family Members in Transcription, Proliferation and Apoptosis. Curr Mol Med. 2006;6(7):739–48. Epub 2006/11/15. . 17100600 10.2174/1566524010606070739

[42] Zhou D, Masri S, Ye JJ, Chen S. Transcriptional regulation of the mouse PNRC2 promoter by the nuclear factor Y (NFY) and E2F1. Gene. 2005;361:89–100. 10.1016/j.gene.2005.07.012 . 16181749

[43] Nicolas M, Noe V, Ciudad CJ. Transcriptional regulation of the human Sp1 gene promoter by the specificity protein (Sp) family members nuclear factor Y (NF-Y) and E2F. The Biochemical journal. 2003;371(Pt 2):265–75. 10.1042/BJ20021166 12513689 PMC1223280

[44] Tabach Y, Milyavsky M, Shats I, Brosh R, Zuk O, Yitzhaky A, et al The promoters of human cell cycle genes integrate signals from two tumor suppressive pathways during cellular transformation. Mol Syst Biol. 2005;1:2005 0022. 10.1038/msb4100030 16729057 PMC1681464

