# Supplementary figures and images for: E2F1-Mediated Induction of NFYB Attenuates Apoptosis via Joint Regulation of a Pro-Survival Transcriptional Program
Source: PLoS One. 2015 Jun 3;10(6):e0127951. doi: 10.1371/journal.pone.0127951 (PMC4454684; doi:10.1371/journal.pone.0127951)

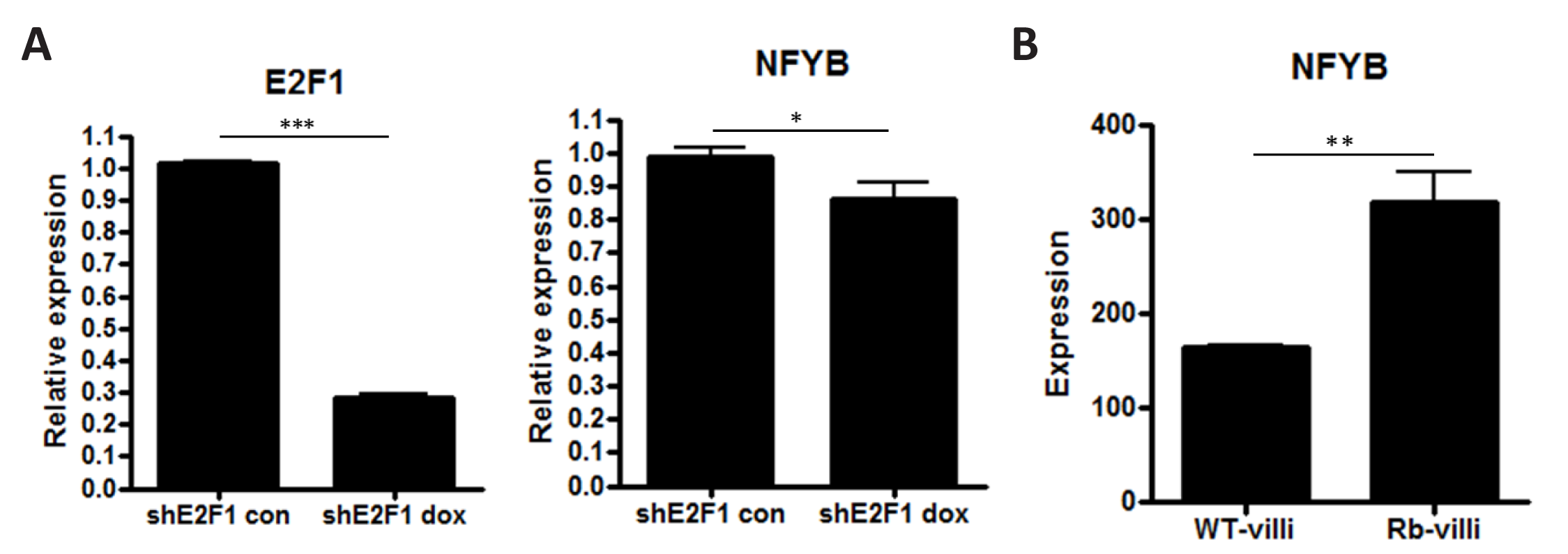

Supplement: S1 Fig — Real time qPCR analysis of E2F1 and NFYB expression following 24 hours doxycyclin-inducible E2F1 knockdown in U2OS cells (A). Publicly available microarray expression data of the intestinal villi from wild type and RB knockout mouse (GSE16454) was analyzed for the expression of NFYB. Data presented are means +/- SD of triplicate samples (reversed logged RMA data for probeset 1419266_at). Asterisks denote statistically significant difference between the two groups as evaluated by t-test (*p<0.05, **p<0.01) (B). (TIF) [file pone.0127951.s001.tif]

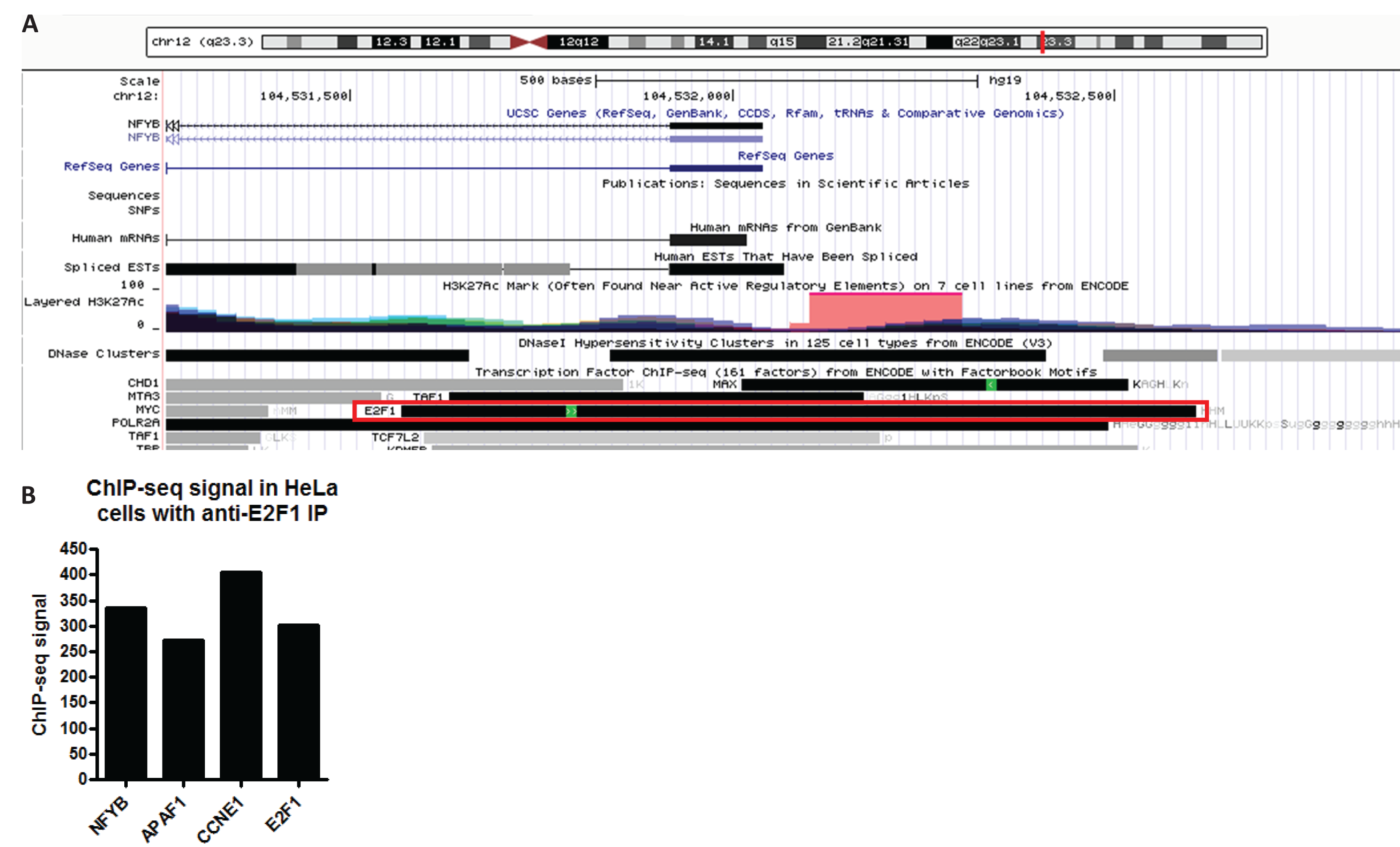

Supplement: S2 Fig — A screen shot of UCSC Genome Browser showing NFYB promoter. Tracks on the bottom represent ChIP-seq signals for different indicated transcription factors with the intensity of black color proportional to the signal strength. Strong binding signal of endogenous E2F1 to the region around the NFYB transcription start site in HeLa cells is boxed (A). The signals of normalized bound reads for E2F1 ChIP-seq in HeLa cells for NFYB, CCNE1, APAF1 and E2F1 promoters were extracted using UCSC Genome Browser and plotted (B). (TIF) [file pone.0127951.s002.tif]

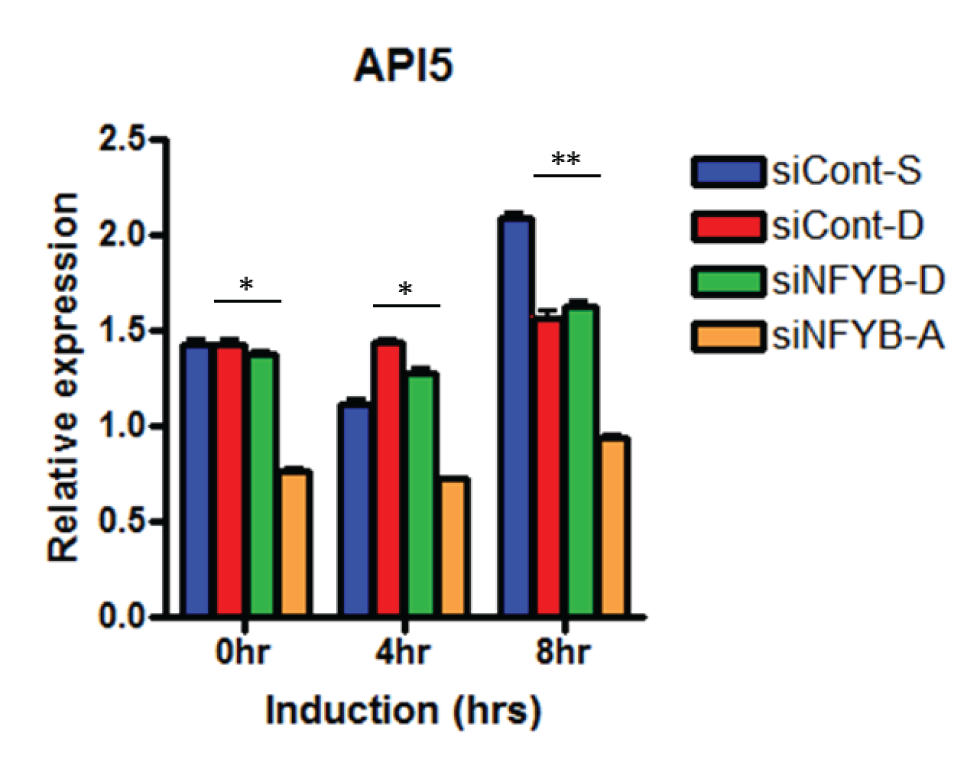

Supplement: S3 Fig — Real-time PCR validation of target gene expression decrease for API5. Samples were processed in the same manner as Fig 2A. * denotes p<0.05, ** denotes p<0.01. (TIF) [file pone.0127951.s003.tif]

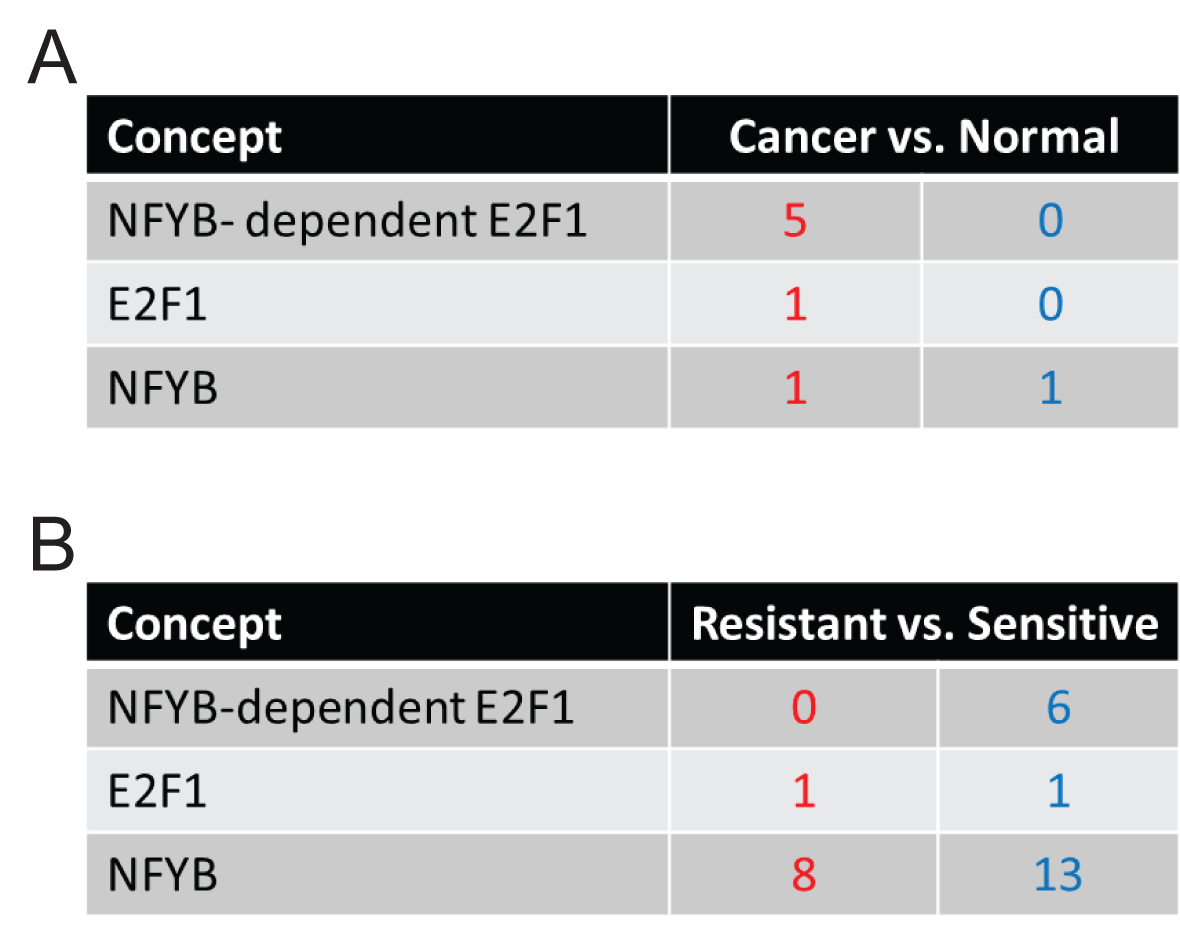

Supplement: S4 Fig — Number of sarcoma datasets from cancer vs normal Oncomine analysis in which signatures (concepts) consisting of differentially expressed genes are significantly associated with the indicated signatures. The NFYB-dependent E2F1 signature (Fig 2D and S2 Table) is derived from our microarray analysis and described in the Results section. The E2F1 (U2OS induced) signature is a concept defined by Oncomine based on previously published microarray analysis of genes upregulated in U2OS ER-E2F1 cells following OHT induction. The E2F1-independent NFYB signature consists of probes (174) which expression is reduced 30% following NFYB knockdown regardless of the effect of E2F1 induction. Significance is set at an odds ratio of at least two and p value of less than 0.0001. Red indicates number of signatures overexpressed in cancer compared to normal cells. Blue represents number of signatures underexpressed in cancer compared to normal (A). Number of datasets in which concepts from therapy sensitivity datasets are significantly associated with the signatures described in (A in S4 Fig). Significance is set at an odds ratio of at least two and p value of less than 0.0001. Red indicates number of signatures overexpressed in drug resistant compared to drug sensitive cells. Blue represents number of signatures underexpressed in resistant compared to sensitive cells (B). (TIF) [file pone.0127951.s004.tif]
