# Supplementary material for: E2F1-Mediated Induction of NFYB Attenuates Apoptosis via Joint Regulation of a Pro-Survival Transcriptional Program
Source: PLoS One. 2015 Jun 3;10(6):e0127951. doi: 10.1371/journal.pone.0127951 (PMC4454684; doi:10.1371/journal.pone.0127951)
Supplement: S1 Table — Unsupervised hierarchical clustering was performed on genes whose expression is significantly different (p<0.001) in E2F1 activated cells following NFYB knockdown by at least 1.3 fold higher or 0.7 fold lower compared to control siRNA, resulting in 5 groups: 1) induced by E2F1 activation and reduced by NFYB knockdown, 2) unaffected by E2F1 activation and reduced by NFYB knockdown, 3) reduced by E2F1 activation and further reduced by NFYB knockdown, 4) induced by E2F1 activation and further induced by NFYB knockdown, 5) reduced by E2F1 but higher in NFYB knockdown. (PDF) [file pone.0127951.s005.pdf]

Cluster 1

Probe.Set.ID	Symbol	Gene Title
201625_s_at	INSIG1	insulin induced gene 1
202036_s_at	SFRP1	secreted frizzled-related protein 1
202037_s_at	SFRP1	secreted frizzled-related protein 1
202178_at	PRKCZ	protein kinase C, zeta
202460_s_at	LPIN2	lipin 2
202842_s_at	DNAJB9	DnaJ (Hsp40) homolog, subfamily B, member 9
202948_at	IL1R1	interleukin 1 receptor, type I
203072_at	MYO1E	myosin IE
203429_s_at	SUCO	chromosome 1 open reading frame 9
203689_s_at	FMR1	fragile X mental retardation 1
204062_s_at	ULK2	unc-51-like kinase 2 (C. elegans)
204160_s_at	ENPP4	ectonucleotide pyrophosphatase/phosphodiesterase 4 (putative function)
204479_at	OSTF1	osteoclast stimulating factor 1
204720_s_at	DNAJC6	DnaJ (Hsp40) homolog, subfamily C, member 6
205359_at	AKAP6	A kinase (PRKA) anchor protein 6
206233_at	B4GALT6	UDP-Gal:betaGlcNAc beta 1,4- galactosyltransferase, polypeptide 6
206615_s_at	ADAM22	ADAM metallopeptidase domain 22
207362_at	SLC30A4	solute carrier family 30 (zinc transporter), member 4
208925_at	CLDND1	claudin domain containing 1
208962_s_at	FADS1	fatty acid desaturase 1
208963_x_at	FADS1	fatty acid desaturase 1
208964_s_at	FADS1	fatty acid desaturase 1
209569_x_at	NSG1	Neuron specific gene family member 1
209925_at	OCLN	occludin pseudogene; occludin
209993_at	ABCB1	ATP-binding cassette, sub-family B (MDR/TAP), member 1
210355_at	PTHLH	parathyroid hormone-like hormone
210829_s_at	SSBP2	single-stranded DNA binding protein 2
210941_at	PCDH7	protocadherin 7
211130_x_at	EDA	ectodysplasin A
211478_s_at	DPP4	dipeptidyl-peptidase 4
212812_at	SERINC5	serine incorporator 5
212870_at	SOS2	son of sevenless homolog 2 (Drosophila)
212959_s_at	GNPTAB	N-acetylglucosamine-1-phosphate transferase, alpha and beta subunits
214023_x_at	TUBB2B	tubulin, beta 2B
214578_s_at	ROCK1	similar to Rho-associated, coiled-coil containing protein kinase 1; Rho-associated, coiled-coil containing protein kinase 1
215245_x_at	FMR1	fragile X mental retardation 1
215363_x_at	FOLH1	folate hydrolase (prostate-specific membrane antigen) 1
215446_s_at	LOX	lysyl oxidase
216167_at	LRRN2	leucine rich repeat neuronal 2
216205_s_at	MFN2	mitofusin 2
216255_s_at	GRM8	glutamate receptor, metabotropic 8
216256_at	GRM8	glutamate receptor, metabotropic 8
218127_at	NFYB	nuclear transcription factor Y, beta
218128_at	NFYB	nuclear transcription factor Y, beta

218129_s_at	NFYB	nuclear transcription factor Y, beta
218223_s_at	PLEKHO1	pleckstrin homology domain containing, family O member 1
219526_at	C14orf169	chromosome 14 open reading frame 169
219703_at	MNS1	meiosis-specific nuclear structural 1
219932_at	SLC27A6	solute carrier family 27 (fatty acid transporter), member 6
221761_at	ADSS	adenylosuccinate synthase
222071_s_at	SLCO4C1	solute carrier organic anion transporter family, member 4C1

Cluster 2

Probe.Set.ID	Symbol	Gene Title
200927_s_at	RAB14	RAB14, member RAS oncogene family
201063_at	RCN1	reticulocalbin 1, EF-hand calcium binding domain
201490_s_at	PPIF	peptidylprolyl isomerase F
201662_s_at	ACSL3	acyl-CoA synthetase long-chain family member 3
201761_at	MTHFD2	methylenetetrahydrofolate dehydrogenase (NADP+ dependent) 2, methenyltetrahydrofolate cyclohydrolase
202101_s_at	RALB	v-ral simian leukemia viral oncogene homolog B (ras related; GTP binding protein)
202516_s_at	DLG1	discs, large homolog 1 (Drosophila)
203548_s_at	LPL	lipoprotein lipase
203688_at	PKD2	polycystic kidney disease 2 (autosomal dominant)
204298_s_at	LOX	lysyl oxidase
205573_s_at	SNX7	sorting nexin 7
206491_s_at	NAPA	N-ethylmaleimide-sensitive factor attachment protein, alpha
206683_at	ZNF165	zinc finger protein 165
208309_s_at	MALT1	mucosa associated lymphoid tissue lymphoma translocation gene 1
208703_s_at	APLP2	amyloid beta (A4) precursor-like protein 2
209232_s_at	DCTN5	dynactin 5 (p25)
209706_at	NKX3-1	NK3 homeobox 1
211048_s_at	PDIA4	protein disulfide isomerase family A, member 4
211804_s_at	CDK2	cyclin-dependent kinase 2
213369_at	CDHR1	protocadherin 21
214959_s_at	API5	API5-like 1; apoptosis inhibitor 5
215489_x_at	HOMER3	homer homolog 3 (Drosophila)
217761_at	ADI1	acireductone dioxygenase 1
217931_at	CNPY3	canopy 3 homolog (zebrafish)
218095_s_at	TMEM165	transmembrane protein 165
219628_at	ZMAT3	zinc finger, matrin type 3
221318_at	NEUROD4	neurogenic differentiation 4
221781_s_at	DNAJC10	DnaJ (Hsp40) homolog, subfamily C, member 10

Cluster 3

Probe.Set.ID	Symbol	Gene Title
201147_s_at	TIMP3	TIMP metallopeptidase inhibitor 3
201341_at	ENC1	ectodermal-neural cortex (with BTB-like domain)
201399_s_at	TRAM1	translocation associated membrane protein 1
201633_s_at	CYB5B	cytochrome b5 type B (outer mitochondrial membrane)

201852_x_at	COL3A1	collagen, type III, alpha 1
201860_s_at	PLAT	plasminogen activator, tissue
201932_at	LRRC41	leucine rich repeat containing 41
202043_s_at	SMS	spermine synthase; similar to spermine synthase
202061_s_at	SEL1L	sel-1 suppressor of lin-12-like (C. elegans)
202062_s_at	SEL1L	sel-1 suppressor of lin-12-like (C. elegans)
202705_at	CCNB2	cyclin B2
202816_s_at	SS18	synovial sarcoma translocation, chromosome 18
202920_at	ANK2	ankyrin 2, neuronal
203041_s_at	LAMP2	lysosomal-associated membrane protein 2
203069_at	SV2A	synaptic vesicle glycoprotein 2A
203179_at	GALT	galactose-1-phosphate uridylyltransferase
203211_s_at	MTMR2	myotubularin related protein 2
203441_s_at	CDH2	cadherin 2, type 1, N-cadherin (neuronal)
203738_at	C5orf22	chromosome 5 open reading frame 22
203827_at	WIPI1	WD repeat domain, phosphoinositide interacting 1
204149_s_at	GSTM4	glutathione S-transferase mu 4
204256_at	ELOVL6	ELOVL family member 6, elongation of long chain fatty acids (FEN1/Elo2, SUR4/Elo3-like, yeast)
204641_at	NEK2	NIMA (never in mitosis gene a)-related kinase 2
204725_s_at	NCK1	NCK adaptor protein 1
204749_at	NAP1L3	nucleosome assembly protein 1-like 3
205523_at	HAPLN1	hyaluronan and proteoglycan link protein 1
205717_x_at	PCDHGC3	protocadherin gamma subfamily C, 3; protocadherin gamma subfamily C, 5; protocadherin gamma subfamily C, 4; protocadherin gamma subfamily A, 12
205822_s_at	HMGCS1	3-hydroxy-3-methylglutaryl-Coenzyme A synthase 1 (soluble)
206369_s_at	PIK3CG	phosphoinositide-3-kinase, catalytic, gamma polypeptide
206463_s_at	DHRS2	dehydrogenase/reductase (SDR family) member 2
207714_s_at	SERPINH1	serpin peptidase inhibitor, clade H (heat shock protein 47), member 1, (collagen binding protein 1)
208180_s_at	HIST1H4H	histone cluster 1, H4l; histone cluster 1, H4k; histone cluster 4, H4; histone cluster 1, H4h; histone cluster 1, H4j; histone cluster 1, H4i; histone cluster 1, H4d; histone cluster 1, H4c; histone cluster 1, H4f; histone cluster 1, H4e; histone cluster 1, H4b; histone cluster 1, H4a; histone cluster 2, H4a; histone cluster 2, H4b
208861_s_at	ATRX	alpha thalassemia/mental retardation syndrome X-linked (RAD54 homolog, S. cerevisiae)
209079_x_at	PCDHGC3	protocadherin gamma subfamily C, 3; protocadherin gamma subfamily C, 5; protocadherin gamma subfamily C, 4; protocadherin gamma subfamily A, 12
209118_s_at	TUBA1A	tubulin, alpha 1a
209198_s_at	SYT11	synaptotagmin XI
209208_at	MPDU1	mannose-P-dolichol utilization defect 1
209234_at	KIF1B	kinesin family member 1B
209295_at	TNFRSF10B	tumor necrosis factor receptor superfamily, member 10b
209550_at	NDN	necdin homolog (mouse)
209846_s_at	BTN3A2	butyrophilin, subfamily 3, member A2

210406_s_at	RAB6C	RAB6C, member RAS oncogene family; RAB6A, member RAS oncogene family; hypothetical LOC100130819; RAB6C-like
210868_s_at	ELOVL6	ELOVL family member 6, elongation of long chain fatty acids (FEN1/Elo2, SUR4/Elo3-like, yeast)
210970_s_at	IBTK	inhibitor of Bruton agammaglobulinemia tyrosine kinase
211063_s_at	NCK1	NCK adaptor protein 1
211066_x_at	PCDHGC3	protocadherin gamma subfamily C, 3; protocadherin gamma subfamily C, 5; protocadherin gamma subfamily C, 4; protocadherin gamma subfamily A, 12
211080_s_at	NEK2	NIMA (never in mitosis gene a)-related kinase 2
211162_x_at	SCD	stearoyl-CoA desaturase (delta-9-desaturase)
211959_at	IGFBP5	insulin-like growth factor binding protein 5
212274_at	LPIN1	lipin 1
212403_at	UBE3B	ubiquitin protein ligase E3B
212613_at	BTN3A2	butyrophilin, subfamily 3, member A2
213645_at	ENOSF1	enolase superfamily member 1
214079_at	DHRS2	dehydrogenase/reductase (SDR family) member 2
214642_x_at	MAGEA5	melanoma antigen family A, 5
214710_s_at	CCNB1	cyclin B1
214945_at	FAM153B	family with sequence similarity 153, member B
215076_s_at	COL3A1	collagen, type III, alpha 1
215836_s_at	PCDHGC3	protocadherin gamma subfamily C, 3; protocadherin gamma subfamily C, 5; protocadherin gamma subfamily C, 4; protocadherin gamma subfamily A, 12
217188_s_at	C14orf1	chromosome 14 open reading frame 1
217749_at	COPG1	coatomer protein complex, subunit gamma
217977_at	MSRB1	selenoprotein X, 1
218047_at	OSBPL9	oxysterol binding protein-like 9
218124_at	RETSAT	retinol saturase (all-trans-retinol 13,14-reductase)
218150_at	ARL5A	ADP-ribosylation factor-like 5A
218396_at	VPS13C	vacuolar protein sorting 13 homolog C (S. cerevisiae)
218539_at	FBXO34	F-box protein 34
219049_at	CSGALNACT1	chondroitin sulfate N-acetylgalactosaminyltransferase 1
219730_at	MED18	mediator complex subunit 18
220205_at	TPTE	transmembrane phosphatase with tensin homology
221730_at	COL5A2	collagen, type V, alpha 2
222209_s_at	TMEM135	transmembrane protein 135

Cluster 4

Probe.Set.ID	Symbol	Gene Title
201419_at	BAP1	BRCA1 associated protein-1 (ubiquitin carboxy-terminal hydrolase)
201565_s_at	ID2	inhibitor of DNA binding 2, dominant negative helix-loop-helix protein
203489_at	SIVA1	SIVA1, apoptosis-inducing factor
204621_s_at	NR4A2	nuclear receptor subfamily 4, group A, member 2
204770_at	TAP2	transporter 2, ATP-binding cassette, sub-family B (MDR/TAP)
204790_at	SMAD7	SMAD family member 7
206565_x_at	GUSBP3	glucuronidase, beta pseudogene
208366_at	PCDH11X	protocadherin 11 X-linked
209478_at	STRA13	stimulated by retinoic acid 13 homolog (mouse)

210792_x_at	SIVA1	SIVA1, apoptosis-inducing factor
213089_at		ENSG00000219982
213147_at	HOXA10	homeobox A10
213577_at	SQLE	squalene epoxidase
215043_s_at	GUSBP3	glucuronidase, beta pseudogene
215599_at	GUSBP3	glucuronidase, beta pseudogene
218350_s_at	GMNN	geminin, DNA replication inhibitor
218642_s_at	CHCHD7	coiled-coil-helix-coiled-coil-helix domain containing 7
220097_s_at	TMEM104	transmembrane protein 104
220983_s_at	NEAT1	sprouty homolog 4 (Drosophila)

Cluster 5

Probe.Set.ID	Symbol	Gene Title
201774_s_at	NCAPD2	non-SMC condensin I complex, subunit D2
202215_s_at	NFYC	nuclear transcription factor Y, gamma
202240_at	PLK1	polo-like kinase 1 (Drosophila)
202736_s_at	LSM4	LSM4 homolog, U6 small nuclear RNA associated (S. cerevisiae)
203478_at	NDUFC1	NADH dehydrogenase (ubiquinone) 1, subcomplex unknown, 1, 6kDa
203709_at	PHKG2	phosphorylase kinase, gamma 2 (testis)
203719_at	ERCC1	excision repair cross-complementing rodent repair deficiency, complementation group 1 (includes overlapping antisense sequence)
203848_at	AKAP8	A kinase (PRKA) anchor protein 8
203880_at	COX17	COX17 cytochrome c oxidase assembly homolog (S. cerevisiae)
203931_s_at	MRPL12	mitochondrial ribosomal protein L12
203980_at	FABP4	fatty acid binding protein 4, adipocyte
204107_at	NFYA	nuclear transcription factor Y, alpha
204108_at	NFYA	nuclear transcription factor Y, alpha
204109_s_at	NFYA	nuclear transcription factor Y, alpha
204218_at	ANAPC15	chromosome 11 open reading frame 51
204475_at	MMP1	matrix metallopeptidase 1 (interstitial collagenase)
205167_s_at	CDC25C	cell division cycle 25 homolog C (S. pombe)
205730_s_at	ABLIM3	actin binding LIM protein family, member 3
205828_at	MMP3	matrix metallopeptidase 3 (stromelysin 1, progelatinase)
206785_s_at	KLRC1	killer cell lectin-like receptor subfamily C, member 1
209223_at	NDUFA2	NADH dehydrogenase (ubiquinone) 1 alpha subcomplex, 2, 8kDa
209682_at	CBLB	Cas-Br-M (murine) ecotropic retroviral transforming sequence b
213322_at	OARD1	chromosome 6 open reading frame 130
213893_x_at	PMS2P2	PMS2 postmeiotic segregation increased 2 (S. cerevisiae)-like
213897_s_at	MRPL23	mitochondrial ribosomal protein L23
214073_at	CTTN	cortactin
214110_s_at	LOC654342	lymphocyte-specific protein 1 pseudogene
214756_x_at	PMS2P1	postmeiotic segregation increased 2-like 1 pseudogene
215293_s_at	PGAP2	post-GPI attachment to proteins 2
215380_s_at	GGCT	gamma-glutamyl cyclotransferase
215498_s_at	MAP2K3	mitogen-activated protein kinase kinase 3
215499_at	MAP2K3	mitogen-activated protein kinase kinase 3
215734_at	IZUMO4	chromosome 19 open reading frame 36

216852_x_at	IGLJ3	immunoglobulin lambda joining 3
217010_s_at	CDC25C	cell division cycle 25 homolog C (S. pombe)
217485_x_at	PMS2P1	postmeiotic segregation increased 2-like 1 pseudogene
217907_at	MRPL18	mitochondrial ribosomal protein L18
218261_at	AP1M2	adaptor-related protein complex 1, mu 2 subunit
218597_s_at	CISD1	CDGSH iron sulfur domain 1
218609_s_at	NUDT2	nudix (nucleoside diphosphate linked moiety X)-type motif 2
218741_at	CENPM	centromere protein M
218774_at	DCPS	decapping enzyme, scavenger
218866_s_at	POLR3K	polymerase (RNA) III (DNA directed) polypeptide K, 12.3 kDa
219053_s_at	VPS37C	vacuolar protein sorting 37 homolog C (S. cerevisiae)
220089_at	L2HGDH	L-2-hydroxyglutarate dehydrogenase
221777_at	RITA1	chromosome 12 open reading frame 52
32259_at	EZH1	enhancer of zeste homolog 1 (Drosophila)
33304_at	ISG20	interferon stimulated exonuclease gene 20kDa
37462_i_at	SF3A2	splicing factor 3a, subunit 2, 66kDa
40569_at	MZF1	myeloid zinc finger 1
41858_at	PGAP2	post-GPI attachment to proteins 2
65517_at	AP1M2	adaptor-related protein complex 1, mu 2 subunit
