# Supplementary material for: E2F1-Mediated Induction of NFYB Attenuates Apoptosis via Joint Regulation of a Pro-Survival Transcriptional Program
Source: PLoS One. 2015 Jun 3;10(6):e0127951. doi: 10.1371/journal.pone.0127951 (PMC4454684; doi:10.1371/journal.pone.0127951)
Supplement: S2 Table — Analysis was performed on microarray data showing 148 genes whose expression is induced at least two fold by E2F1 activation and significantly lower (p<0.05) following NFYB knockdown at a ratio of 1:1.2 compared to control. (PDF) [file pone.0127951.s006.pdf]

Probe.Set.ID	Symbol	Gene Title
200762_at	DPYSL2	dihydropyrimidinase-like 2
201283_s_at	TRAK1	trafficking protein, kinesin binding 1
201688_s_at	TPD52	tumor protein D52
201689_s_at	TPD52	tumor protein D52
201693_s_at	EGR1	early growth response 1
201711_x_at	RANBP2	RAN binding protein 2
202035_s_at	SFRP1	secreted frizzled-related protein 1
202036_s_at	SFRP1	secreted frizzled-related protein 1
202037_s_at	SFRP1	secreted frizzled-related protein 1
202178_at	PRKCZ	protein kinase C, zeta
202236_s_at	SLC16A1	solute carrier family 16 (monocarboxylic acid transporters), member 1
202583_s_at	RANBP9	RAN binding protein 9
202670_at	MAP2K1	mitogen-activated protein kinase kinase 1
202842_s_at	DNAJB9	DnaJ (Hsp40) homolog, subfamily B, member 9
202843_at	DNAJB9	ralA binding protein 1
202948_at	IL1R1	interleukin 1 receptor, type I
203044_at	CHSY1	carbohydrate (chondroitin) synthase 1
203057_s_at	PRDM2	PR domain containing 2, with ZNF domain
203072_at	MYO1E	myosin IE
203311_s_at	ARF6	ADP-ribosylation factor 6
203520_s_at	ZNF318	zinc finger protein 318
203625_x_at	SKP2	S-phase kinase-associated protein 2 (p45)
203641_s_at	COBLL1	COBL-like 1
203689_s_at	FMR1	fragile X mental retardation 1
203935_at	ACVR1	activin A receptor, type I
204062_s_at	ULK2	unc-51-like kinase 2 (C. elegans)
204145_at	FRG1	FSHD region gene 1
204160_s_at	ENPP4	ectonucleotide pyrophosphatase/phosphodiesterase 4 (putative function)
204161_s_at	ENPP4	ectonucleotide pyrophosphatase/phosphodiesterase 4 (putative function)
204184_s_at	ADRBK2	adrenergic, beta, receptor kinase 2
204194_at	BACH1	BTB and CNC homology 1, basic leucine zipper transcription factor 1
204249_s_at	LMO2	LIM domain only 2 (rhombotin-like 1)
204392_at	CAMK1	calcium/calmodulin-dependent protein kinase I
204479_at	OSTF1	osteoclast stimulating factor 1
204497_at	ADCY9	adenylate cyclase 9
204507_s_at	PPP3R1	protein phosphatase 3 (formerly 2B), regulatory subunit B, 19kDa, alpha isoform (calcineurin B, type I)
204526_s_at	TBC1D8	TBC1 domain family, member 8 (with GRAM domain)
204720_s_at	DNAJC6	DnaJ (Hsp40) homolog, subfamily C, member 6
204748_at	PTGS2	prostaglandin-endoperoxide synthase 2 (prostaglandin G/H synthase and cyclooxygenase)
204832_s_at	BMPR1A	bone morphogenetic protein receptor, type IA
204953_at	SNAP91	synaptosomal-associated protein, 91kDa homolog (mouse)
204984_at	GPC4	glypican 4
204989_s_at	ITGB4	integrin, beta 4
205015_s_at	TGFA	transforming growth factor, alpha

205016_at	TGFA	transforming growth factor, alpha
205123_s_at	TMEFF1	transmembrane protein with EGF-like and two follistatin-like domains 1
205164_at	GCAT	glycine C-acetyltransferase (2-amino-3-ketobutyrate coenzyme A ligase)
205333_s_at	RCE1	RCE1 homolog, prenyl protein peptidase (S. cerevisiae)
205462_s_at	HPCAL1	hippocalcin-like 1
205478_at	PPP1R1A	protein phosphatase 1, regulatory (inhibitor) subunit 1A
205789_at	CD1D	CD1d molecule /// CD1d molecule
205842_s_at	JAK2	Janus kinase 2 (a protein tyrosine kinase)
205850_s_at	GABRB3	gamma-aminobutyric acid (GABA) A receptor, beta 3
205860_x_at	FOLH1	folate hydrolase (prostate-specific membrane antigen) 1
206159_at	GDF10	growth differentiation factor 10
206176_at	BMP6	bone morphogenetic protein 6
206233_at	B4GALT6	UDP-Gal:betaGlcNAc beta 1,4- galactosyltransferase, polypeptide 6
206456_at	GABRA5	gamma-aminobutyric acid (GABA) A receptor, alpha 5
206511_s_at	SIX2	sine oculis homeobox homolog 2 (Drosophila)
206615_s_at	ADAM22	ADAM metallopeptidase domain 22
207150_at	SLC18A3	solute carrier family 18 (vesicular acetylcholine), member 3
207265_s_at	KDELR3	KDEL (Lys-Asp-Glu-Leu) endoplasmic reticulum protein retention receptor 3
207275_s_at	ACSL1	acyl-CoA synthetase long-chain family member 1
207362_at	SLC30A4	solute carrier family 30 (zinc transporter), member 4
207767_s_at	EGR4	early growth response 4
207768_at	EGR4	early growth response 4
207781_s_at	ZNF711	zinc finger protein 711
207824_s_at	MAZ	MYC-associated zinc finger protein (purine-binding transcription factor)
208237_x_at	ADAM22	ADAM metallopeptidase domain 22
208244_at	BMP3	bone morphogenetic protein 3 (osteogenic)
208264_s_at	EIF3J	eukaryotic translation initiation factor 3, subunit 1 alpha, 35kDa
208606_s_at	WNT4	wingless-type MMTV integration site family, member 4 /// wingless-type MMTV integration site family, member 4
208652_at	PPP2CA	protein phosphatase 2 (formerly 2A), catalytic subunit, alpha isoform
208985_s_at	EIF3J	eukaryotic translation initiation factor 3, subunit 1 alpha, 35kDa
208990_s_at	HNRNPH3	heterogeneous nuclear ribonucleoprotein H3 (2H9)
209098_s_at	JAG1	jagged 1 (Alagille syndrome)
209112_at	CDKN1B	cyclin-dependent kinase inhibitor 1B (p27, Kip1)
209281_s_at	ATP2B1	ATPase, Ca++ transporting, plasma membrane 1
209339_at	SIAH2	seven in absentia homolog 2 (Drosophila) /// seven in absentia homolog 2 (Drosophila)
209347_s_at	MAF	v-maf musculoaponeurotic fibrosarcoma oncogene homolog (avian)
209569_x_at	NSG1	Neuron specific gene family member 1
209570_s_at	NSG1	Neuron specific gene family member 1
209590_at	BMP7	Bone morphogenetic protein 7 (osteogenic protein 1)
209591_s_at	BMP7	bone morphogenetic protein 7 (osteogenic protein 1)
209990_s_at	GABBR2	gamma-aminobutyric acid (GABA) B receptor, 2
210021_s_at	CCNO	uracil-DNA glycosylase 2
210127_at	RAB6B	RAB6B, member RAS oncogene family
210190_at	STX11	syntaxin 11
210240_s_at	CDKN2D	cyclin-dependent kinase inhibitor 2D (p19, inhibits CDK4)

210355_at	PTHLH	parathyroid hormone-like hormone
210447_at	GLUD2	glutamate dehydrogenase 2
210480_s_at	MYO6	myosin VI
210540_s_at	B4GALT4	UDP-Gal:betaGlcNAc beta 1,4- galactosyltransferase, polypeptide 4
210555_s_at	NFATC3	nuclear factor of activated T-cells, cytoplasmic, calcineurin-dependent 3
210716_s_at	CLIP1	restin (Reed-Steinberg cell-expressed intermediate filament-associated protein)
210829_s_at	SSBP2	single-stranded DNA binding protein 2
210875_s_at	ZEB1	transcription factor 8 (represses interleukin 2 expression)
211067_s_at	GAS7	growth arrest-specific 7 /// growth arrest-specific 7
211171_s_at	PDE10A	phosphodiesterase 10A
211379_x_at	B3GALNT1	UDP-GalNAc:betaGlcNAc beta 1,3-galactosaminyltransferase, polypeptide 1 (Globoside blood group)
211478_s_at	DPP4	dipeptidyl-peptidase 4 (CD26, adenosine deaminase complexing protein 2)
211631_x_at	B4GALT1	UDP-Gal:betaGlcNAc beta 1,4- galactosyltransferase, polypeptide 1
211812_s_at	B3GALNT1	UDP-GalNAc:betaGlcNAc beta 1,3-galactosaminyltransferase, polypeptide 1 (Globoside blood group)
211985_s_at	CALM1	calmodulin 1 (phosphorylase kinase, delta)
212056_at	GSE1	KIAA0182
212209_at	MED13L	thyroid hormone receptor associated protein 2
212435_at	TRIM33	tripartite motif-containing 33
212447_at	KBTBD2	kelch repeat and BTB (POZ) domain containing 2
212521_s_at	PDE8A	phosphodiesterase 8A
212750_at	PPP1R16B	protein phosphatase 1, regulatory (inhibitor) subunit 16B
212812_at	SERINC5	Serine incorporator 5
212870_at	SOS2	Ras association (RalGDS/AF-6) domain family 3
212930_at	ATP2B1	ATPase, Ca++ transporting, plasma membrane 1
212986_s_at	TLK2	tousled-like kinase 2
213353_at	ABCA5	ATP-binding cassette, sub-family A (ABC1), member 5
213469_at	PGAP1	GPI deacylase
213470_s_at	HNRNPH1	heterogeneous nuclear ribonucleoprotein H1 (H)
213533_at	NSG1	Neuron specific gene family member 1
213695_at	PON3	paraoxonase 3
213906_at	MYBL1	v-myb myeloblastosis viral oncogene homolog (avian)-like 1
214449_s_at	RHOQ	ras homolog gene family, member Q
214543_x_at	QKI	quaking homolog, KH domain RNA binding (mouse)
214578_s_at	ROCK1	similar to Rho-associated protein kinase 1 (Rho-associated, coiled-coil containing protein kinase 1) (p160 ROCK-1) (p160ROCK)
214691_x_at	FAM63B	family with sequence similarity 63, member B
214790_at	SENP6	SUMO1/sentrin specific peptidase 6
214890_s_at	FAM149A	DKFZP564J102 protein
214954_at	SUSD5	sushi domain containing 5
215245_x_at	FMR1	fragile X mental retardation 1
215363_x_at	FOLH1	folate hydrolase (prostate-specific membrane antigen) 1
215716_s_at	ATP2B1	ATPase, Ca++ transporting, plasma membrane 1
215794_x_at	GLUD1	glutamate dehydrogenase 2
216125_s_at	RANBP9	RAN binding protein 9

216255_s_at	GRM8	glutamate receptor, metabotropic 8
216256_at	GRM8	glutamate receptor, metabotropic 8
216350_s_at	ZNF10	zinc finger protein 10
216488_s_at	ATP11A	ATPase, Class VI, type 11A
216521_s_at	BRCC3	BRCA1/BRCA2-containing complex, subunit 3
216627_s_at	B4GALT1	UDP-Gal:betaGlcNAc beta 1,4- galactosyltransferase, polypeptide 1
216870_x_at	DLEU2	deleted in lymphocytic leukemia, 2
216953_s_at	WT1	Wilms tumor 1
217280_x_at	GABRA5	gamma-aminobutyric acid (GABA) A receptor, alpha 5
217644_s_at	SOS2	son of sevenless homolog 2 (Drosophila)
217920_at	MAN1A2	mannosidase, alpha, class 1A, member 2
218127_at	NFYB	nuclear transcription factor Y, beta
218128_at	NFYB	nuclear transcription factor Y, beta
218129_s_at	NFYB	nuclear transcription factor Y, beta
218182_s_at	CLDN1	claudin 1
218223_s_at	PLEKHO1	pleckstrin homology domain containing, family O member 1
218319_at	PELI1	pellino homolog 1 (Drosophila)
218499_at	MST4	Mst3 and SOK1-related kinase
219312_s_at	ZBTB10	zinc finger and BTB domain containing 10
219631_at	LRP12	low density lipoprotein-related protein 12
219703_at	MNS1	meiosis-specific nuclear structural 1
219778_at	ZFPM2	zinc finger protein, multitype 2
219797_at	MGAT4A	mannosyl (alpha-1,3-)-glycoprotein beta-1,4-N-acetylglucosaminyltransferase, isozyme A
219864_s_at	RCAN3	Down syndrome critical region gene 1-like 2
219892_at	TM6SF1	transmembrane 6 superfamily member 1
219932_at	SLC27A6	solute carrier family 27 (fatty acid transporter), member 6
220014_at	PRR16	mesenchymal stem cell protein DSC54
220120_s_at	EPB41L4A	erythrocyte membrane protein band 4.1 like 4A
220253_s_at	LRP12	low density lipoprotein-related protein 12
220254_at	LRP12	low density lipoprotein-related protein 12
220265_at	GPR107	G protein-coupled receptor 107
220386_s_at	EML4	echinoderm microtubule associated protein like 4
220955_x_at	RAB23	RAB23, member RAS oncogene family
221039_s_at	ASAP1	development and differentiation enhancing factor 1
221268_s_at	SGPP1	sphingosine-1-phosphate phosphatase 1 /// sphingosine-1-phosphate phosphatase 1
221428_s_at	TBL1XR1	transducin (beta)-like 1X-linked receptor 1 /// transducin (beta)-like 1X-linked receptor 1
221814_at	GPR124	G protein-coupled receptor 124
222071_s_at	SLCO4C1	solute carrier organic anion transporter family, member 4C1
222121_at	ARHGEF26	Src homology 3 domain-containing guanine nucleotide exchange factor
222235_s_at	CSGALNACT2	chondroitin sulfate GalNAcT-2
41577_at	PPP1R16B	protein phosphatase 1, regulatory (inhibitor) subunit 16B
59644_at	BMP2K	BMP2 inducible kinase
